# The wheat AGC kinase TaAGC1 is a positive contributor to host resistance to the necrotrophic pathogen *Rhizoctonia cerealis*

**DOI:** 10.1093/jxb/erv367

**Published:** 2015-07-27

**Authors:** Xiuliang Zhu, Kun Yang, Xuening Wei, Qiaofeng Zhang, Wei Rong, Lipu Du, Xingguo Ye, Lin Qi, Zengyan Zhang

**Affiliations:** ^1^The National Key Facility for Crop Gene Resources and Genetic Improvement, Key Laboratory of Biology and Genetic Improvement of Triticeae Crops, Ministry of Agriculture, Institute of Crop Science, Chinese Academy of Agricultural Sciences, Beijing 100081, China; ^2^Jiangsu Academy of Agricultural Sciences, Nanjing 210014, China

**Keywords:** AGC kinase, differential expression, reactive oxygen species, resistance, *Rhizoctonia cerealis*, *Triticum aestivum*, wheat.

## Abstract

Considerable progress has been made in understanding the roles of AGC kinases in mammalian systems. However, very little is known about the roles of AGC kinases in wheat (*Triticum aestivum*). The necrotrophic fungus *Rhizoctonia cerealis* is the major pathogen of the destructive disease sharp eyespot of wheat. In this study, the wheat AGC kinase gene *TaAGC1*, responding to *R. cerealis* infection, was isolated, and its properties and role in wheat defence were characterized. *R. cerealis*-resistant wheat lines expressed *TaAGC1* at higher levels than susceptible wheat lines. Sequence and phylogenetic analyses showed that the TaAGC1 protein is a serine/threonine kinase belonging to the NDR (nuclear Dbf2-related) subgroup of AGC kinases. Kinase activity assays proved that TaAGC1 is a functional kinase and the Asp-239 residue located in the conserved serine/threonine kinase domain of TaAGC1 is required for the kinase activity. Subcellular localization assays indicated that TaAGC1 localized in the cytoplasm and nucleus. Virus-induced *TaAGC1* silencing revealed that the down-regulation of *TaAGC1* transcripts significantly impaired wheat resistance to *R. cerealis*. The molecular characterization and responses of *TaAGC1* overexpressing transgenic wheat plants indicated that *TaAGC1* overexpression significantly enhanced resistance to sharp eyespot and reduced the accumulation of reactive oxygen species (ROS) in wheat plants challenged with *R*. *cerealis*. Furthermore, ROS-scavenging and certain defence-associated genes were up-regulated in resistant plants overexpressing *TaAGC1* but down-regulated in susceptible knock-down plants. These results suggested that the kinase TaAGC1 positively contributes to wheat immunity to *R*. *cerealis* through regulating expression of ROS-related and defence-associated genes.

## Highlight

The wheat NDR-type AGC kinase TaAGC1 positively regulates host defence response to the necrotrophic phytopathogen Rhizoctonia cerealis through modulating expression of ROS-related and defence-associated genes.

## Introduction

Common wheat (*Triticum aestivum* L.) is a widely cultivated and consumed staple crop, feeding ~35% of the world’s population (Scofield *et al.*, 2005). Sharp eyespot is a devastating disease of wheat globally (Chen *et al.*, 2008, 2013; Hamada *et al.*, 2011; Lemańczyk and Kwaśna, 2013) and since 2005 the epidemic has become severe across large parts of China, with 9.33 m ha of wheat affected by sharp eyespot in 2015 (http://www.agri.cn/V20/bchqb/201501/t20150121_4344729.htm). The main agent of wheat sharp eyespot is *Rhizoctonia cerealis* van der Hoeven, a soil-borne fungus with bi-nucleated hyphal cells (Chen *et al.*, 2013). Sharp eyespot manifests as ‘eye’-shaped lesions on basal sheaths and stems of infected wheat plants. The disease can destroy the transport tissues in stems of plants and block transportation of substances required for nutrition, leading to yield losses of ~10–40%. Breeding resistant wheat varieties is an effective and environmentally safe approach to control disease. However, traditional resistance breeding is difficult because the resistance in wheat accessions is partial and quantitative (Cai *et al.*, 2006; Chen *et al.*, 2013). The wheat defence response to *R. cerealis* is complicated and involves a series of gene expression changes. To improve wheat resistance to sharp eyespot, it is vital to identify genes that play an important role in the defence and unravel their underlying functional mechanisms.

Plant protein kinases, including receptor-like kinases, regulate recognition and early responses to diverse signals, and frequently play critical roles in various developmental and physiological processes, including defence and symbiosis (Rentel *et al.*, 2004; AbuQamar *et al.*, 2008; Fu *et al.*, 2009; Garcia *et al.*, 2012). The AGC kinase [named after cAMP-dependent protein kinase A (PKA), cGMP-dependent protein kinase G (PKG) and phospholipid-dependent protein kinase C (PKC)] family is a group of serine/threonine (Ser/Thr) protein kinases that share sequence similarities in their catalytic kinase domains with PKA, PKG and PKC (Pearce *et al.*, 2010), and is conserved among eukaryotic genomes. In mammalian systems, AGC kinases are important regulators of cell growth, metabolism and cell death (Pearce *et al.*, 2010). Although the catalytic kinase domains of plant AGC kinases share sequence similarities with those of mammalian AGC kinases, little is known about their molecular functions and targets (Garcia *et al.*, 2012). In the *Arabidopsis* genome, there are 39 AGC kinase members (Bogre *et al.*, 2003). Based on a catalytic domain comparison, these AGC kinases were classified into six subfamilies: PDK1, AGC VI, AGC VII (including NDR kinases), AGC VIIIa, AGC VIIIb and ‘other’ (Bogre *et al.*, 2003). To date, in three model plant species (*Arabidopsis*, tomato, and rice), several AGC kinases have been implicated in growth, morphogenesis and defence responses to pathogens (Devarenne *et al.*, 2006; Matsui *et al.*, 2010; Camehl *et al.*, 2011; Garcia *et al.*, 2012). For example, in tomato, an AvrPto-dependent Pto-interacting protein 3 (Adi3), a member of the AGC VIIIb subfamily, is phosphorylated by the resistance kinase Pto and 3-phosphoinositide- dependent protein kinase-1 (Pdk1), and negatively regulates cell death that is associated with *P. syringae* pv. *tomato* (Devarenne *et al.*, 2006). In *Arabidopsis*, OXIDATIVE SIGNAL-INDUCIBLE1 (AtOXI1) is an AGC VIIIb kinase and is required for immunity against the pathogens *Hyaloperonospora arabidopsidis* (an oomycete) and *Pseudomonas syringae* pv. *tomato* DC3000 (a bacterial biotrophic pathogen) (Rentel *et al.*, 2004; Petersen *et al.*, 2009). The rice AGC kinase OsOxi1, an orthologue of AtOXI1, phosphorylates the residue Thr-233 of OsPti1a and then releases OsPti1a-mediated inhibition of disease resistance, resulting in positive contribution to basal resistance to the blast fungus *Magnaporthe oryzae* pv. *oryzae* (Matsui *et al.*, 2010). These AGC kinases belong to the AGC VIIIb subfamily. In different plant species, AGC kinases may have different regulatory roles in response to abiotic stresses. Thus, the isolation and functional study of species-specific AGC kinase genes are important. The large genome size and polyploidy of common wheat has hampered the isolation and functional analysis of wheat genes. So far, in NCBI databases, only seven sequences of common wheat AGC kinases (GenBank accession numbers: CDM81915.1, CDM82518.1, BAF79635.1, BAD19066.1, BAD19068.1, CDM81704.1, CDM85216.1) have been identified by us based on the activation loop signature motif (Bogre *et al.*, 2003) of AGC kinases. However, no functional study of AGC kinase in common wheat has been reported yet.

In early responses to biotic and abiotic stresses, plants often generate an excessive amount of reactive oxygen species (ROS) (Shetty *et al.* 2008; Dong *et al.*, 2013; Schmidt *et al.*, 2013). Necrotroph-induced ROS include superoxide anion (O_2_^−^) and hydrogen peroxide (H_2_O_2_) (Shetty *et al.*, 2008). The levels of several enzymes involved in ROS-scavenging and antioxidative defence, such as superoxide dismutase (SOD), peroxidase (POX), and catalase (CAT), were significantly altered in *Alternaria*-tolerant *Brassica* lines after infection with *Alternaria brassicae* and in tomato infected with *Botrytis cinerea*, indicating that ROS-scavenging enzymes and antioxidants may play significant roles in plant defences against these necrotrophic pathogens (Kuźniak and Skłodowska 2004; Sharma *et al.*, 2007). How the ROS signal is perceived is currently unknown. However, a signal transduction cascade, including the activation of protein kinases and downstream transcription factors, has been proposed in *Arabidopsis* (Mittler *et al.*, 2004; Schmidt *et al.*, 2013). The *Arabidopsis* AGC kinase AtOXI1 is necessary for the ROS signalling pathway (Rentel *et al.*, 2004). However, in wheat, very little is known about the roles of AGC kinases in the regulation of ROS signalling during plant defence responses to necrotrophic pathogens.

In this study, to isolate kinase genes involved in wheat defence response to *R. cerealis*, Agilent wheat microarray chips were used to identify wheat kinase genes expressed differentially between the sharp eyespot-resistant wheat CI12633 and the susceptible wheat Wenmai 6, following infection with *R. cerealis*. We identified the probe corresponding to the 3′-terminal sequence of the wheat AGC kinase gene *TaAGC1*, which showed higher expression in resistant wheat lines (CI12633 and Shanhongmai) than in susceptible wheat line Wenmai 6 inoculated with *R. cerealis*. Then, the *TaAGC1* gene was cloned from resistant wheat line CI12633, and further characterized. The TaAGC1 protein was evidenced to have kinase activity. The role of *TaAGC1* in host defence responses to *R. cerealis* was explored using both *TaAGC1* silencing- and overexpressing- wheat plants. The results showed that TaAGC1, acting as a positive regulator, mediated wheat resistance responses to *R. cerealis.*

## Materials and methods

### Plant and fungal materials and treatments

Five wheat (*T. aestivum*) lines/cultivars—sharp eyespot-resistant CI12633 and Shanhongmai, moderately-susceptible Yangmai 158 and Yangmai 20, and susceptible Wenmai 6 (Supplementary Table S1)—were used in this research. A major Jiangsu virulent isolate of the pathogen *R. cerealis*, R0301, was provided by Profs Huaigu Chen and Shibin Cai of Jiangsu Academy of Agricultural Sciences, China.

Wheat plants were grown in a 14h light/10h dark (23°C/10°C) regime. At the tillering stage, the second base sheath of each wheat plant was inoculated with small toothpick fragments harbouring well-developed mycelia of *R. cerealis*. Inoculated plants were grown at 90% relative humidity for 4 d. The inoculated stems, leaves or roots were sampled at 0, 4, 7 and 21 d post-inoculation (dpi). Additionally, the leaves of CI12633 seedlings at the three-leaf stage were sprayed with 10mM H_2_O_2_ solution for 0, 1, 3, 6 and 12h, following the protocol of Zhu *et al.* (2014). The samples were harvested, frozen quickly in liquid nitrogen, and stored at −80°C prior to total RNA extraction.

### DNA or RNA extraction and cDNA synthesis

Genomic DNA for each sample was isolated from the wheat leaves using the CTAB method (Saghai-Maroof *et al.*, 1984). Total RNA was extracted using TRIzol (Invitrogen), and then subjected to RNase-free DNase I (Promega) digestion and purification. The purified RNA samples (4 µg each) were separately reverse-transcribed to cDNA.

### Microarray hybridizations and data analysis

For each sample total RNA was extracted from the sheaths of 10 seedlings from sharp eyespot-resistant wheat lines (CI12633 and Shanhongmai) or the susceptible wheat cv. Wenmai 6 at 4 or 21 dpi with *R. cerealis.* After purification, the RNA was used for the preparation of Cy5- and Cy3-labelled complementary RNA. These probes of Cy3-labelled CI12633/Shanhongmai RNA and Cy5-labelled Wenmai 6 RNA were hybridized with Agilent Wheat Gene Expression Microarray containing 43 803 probe sets according to the manufacturer’s protocols. The hybridization signals were scanned with an Agilent G2505C Microarray Scanner System, and microarray data were extracted using Feature Extraction Software (v.10.7.1.1) available from Agilent by using the default variables. Data files were loaded into GeneSpring GX 11.5 (Agilent Technologies). The signal values between both experimental groups (CI12633 or Shanhongmai probe vs Wenmai 6 probe) were assessed for each gene based on the normalized probe signals. Differentially expressed kinase genes between CI12633 or Shanhongmai and Yangmai 12 were filtered out by a fold change threshold ≥2.0.

### Isolation and characterization of the *TaAGC1* sequence

The microarray analysis indicated that a probe (TIGR number: TC411796) of the Agilent wheat microarray, the 3′-terminal partial sequence of *TaAGC1* cDNA, was expressed at a significantly higher level in the resistant wheat CI12633 than in the susceptible wheat Wenmai 6. TC411796 was homologous to a wheat AGC kinase gene *WNDr1A* (GenBank accession AB179755) in the National Center for Biotechnology Information (NCBI) database. The 3′-untranslated region (UTR) and full-length open reading frame (ORF) sequences of *TaAGC1* were separately amplified using 3′-Full RACE Core Set kit v.2.0 (TaKaRa) or RT-PCR from cDNA of CI12633 stems inoculated with *R. cerealis* for 4 d. The deduced protein sequence was analysed using the Compute pI/Mw tool (http://web.expasy.org/compute_pi/) to determine the theoretical iso-electric point (pI) and molecular weight (MW), interPro-Scan (http://www.ebi.ac.uk/interpro/) to identify domains and Smart software (http://smart.embl-heidelberg.de/) to predict conserved motifs. A phylogenetic tree was constructed using a neighbor-joining method implemented with MEGA 5.0 software followed by an alignment with other protein kinases using ClustalX software.

### Autophosphorylation and phosphorylation activity assay of TaAGC1 kinase *in vitro*

The residue 239 aspartate (D) at the Ser/Thr kinase active-site of TaAGC1 protein was replaced with alanine (A) to generate the D239A mutant of TaAGC1. To investigate if the TaAGC1 protein is an active kinase and the D239 is important for the kinase activity, the ORF sequence of *TaAGC1* or the D239A mutant was separately subcloned in-frame to the 3′-terminus of a glutathione S-transferase (GST) gene in the pGEX-4T-1 vector (GE Healthcare). The resulting pGST-TaAGC1 and mutant pGST-D239A fusion constructs were transformed into competent cells of *Escherichia coli* BL21 (DE3), respectively. The recombinant GST-TaAGC1 and GST-D239A proteins were separately expressed after treatment with 0.1mM isopropyl-β-D-thiogalactopyranose at 16°C for 19h, and purified using a MicroSpin module (GE Healthcare).

Autophosphorylation and myelin basic protein (MBP) phosphorylation assays were carried out using a reaction mixture (10 μl) consisting of 40mM Hepes-NaOH (pH 8.0), 5mM Mg(CH3COO)_2_, 0.1mM EGTA, 2mM dithiothreitol, 100mM [γ^32^P]ATP and ~100ng of GST-TaAGC1 or GST-D239A. For phosphorylation experiments of protein substrates, 1 μg MBP was incubated with ~100ng of GST-TaAGC1 or GST-D239A under the phosphorylation conditions described above. The reaction was incubated at 30°C for 30min. Then, 2 μl SDS sample buffer was added to the phosphorylation mixture to stop the reaction. The autophosphorylated and phosphorylated proteins were resolved by SDS-PAGE and analysed by autoradiography. After autoradiography, the proteins were stained for 10min by Coomassie Brilliant Blue (CBB) and then photographed.

### Subcellular localization of TaAGC1 in onion and wheat

The coding region of *TaAGC1*, lacking a stop codon, was amplified using the primers *TaAGC1*-GFP-F/*TaAGC1*-GFP-R. The amplified fragment was sub-cloned in-frame with the 5′-terminus of the green fluorescent protein’s (GFP) coding region in the p35S:GFP vector, resulting in the *TaAGC1* fusion construct p35S:TaAGC1-GFP.

The resulting p35S:TaAGC1-GFP and p35S:GFP constructs were separately bombarded into white onion epidermal cells, following the protocol of Zhang *et al.* (2007), or were introduced into wheat mesophyll protoplasts via the PEG-mediated transfection method, following the protocol of Yoo *et al.* (2007). The transformed wheat mesophyll protoplasts or onion epidermal cells were incubated at 25°C for 15h. GFP signals were observed and photographed using a confocal laser scanning microscope (Zeiss LSM 700) with a Fluar 10X/0.50 M27 objective lens and SP640 filter.

### Virus-induced gene silencing assay for TaAGC1 function

The virus-induced gene silencing (VIGS) assay in barley and wheat was developed using the barley stripe mosaic virus (BSMV) (Holzberg *et al.*, 2002). BSMV-VIGS technique is an effective reverse genetic tool for rapidly investigating the functions of genes (Scofield *et al.*, 2005; Zhou *et al.*, 2007; Zhu *et al.*, 2014). To generate the BSMV:TaAGC1 recombinant construct, a 302-bp sequence of TaAGC1 (from 1634 to 1935 nucleotides in TaAGC1 cDNA sequence) was sub-cloned in an antisense orientation into the *NheI* restriction site of the RNAγ of BSMV (Supplementary Fig. S1). Following the protocols described by Holzberg *et al.* (2002) and Zhou *et al.* (2007), the tripartite cDNA chains of BSMV:TaAGC1 or the control BSMV:GFP virus genome were separately transcribed into RNAs, mixed, and used to infect CI12633 plants at the three-leaf stage. At 10 d after infection, the fourth leaves of the inoculated seedlings were collected to monitor BSMV infection, based on the transcription of the BSMV coat protein (*CP*) gene, and to evaluate the transcript changes of *TaAGC1*. At 14 d after BSMV infection, the BSMV-infected CI12633 plants were inoculated with *R. cerealis.* They were scored at 14 and 40 dpi with *R. cerealis*, respectively.

### *TaAGC1*-overexpressing transformation vector and wheat transformation

The *TaAGC1* ORF sequence was amplified with the primers including the *Spe*I and *Sac*I restriction sequences, and then subcloned into the *Spe*I and *Sac*I sites of a modified monocot transformation vector pAHC25 (Christensen and Quail, 1996) with a *c-myc* epitope tag, resulting in the overexpression transformation vector pUBI:myc-TaAGC1. In the construct, the transcript of the myc-TaAGC1 fusion gene was driven by a maize ubiquitin (Ubi) promoter and terminated by 3′ non-transcribed region of *Agrobacterium tumefaciens* nopaline synthase gene (Tnos). A total of 1500 immature embryos of the wheat cultivar Yangmai 20 were transformed by biolistic bombardment using pUBI:myc-TaAGC1 following the protocol described by Chen *et al.* (2008).

### PCR and western blot analyses on *TaAGC1*-overexpressing transgenic wheat

The presence of the *TaAGC1*-overexpressing transgene in the transformed wheat plants was monitored by PCR using the specific primers, TaAGC1-1584F (located in *TaAGC1* coding sequence) and Tnos-R (in Tnos of the transformation vector). PCR was performed in a 20-μl volume containing 200ng genomic DNA, 1×PCR buffer (TaKaRa), 0.4 μM each primer, 200 μM each dNTP and 1U Taq polymerase (TaKaRa). The amplified product (386bp) specific to the introduced TaAGC1-Tnos chimera was resolved on a 1.5% agarose gel and visualized by ethidium bromide staining.

The myc-TaAGC1 fusion protein in the overexpressing transgenic wheat lines was visualized by western blotting analysis. Total proteins were extracted from 0.3g of ground leaf powder. Total soluble proteins (~12 μg) for each line were separated on 12% SDS-PAGE and transferred to polyvinyl difluoride membranes (Amersham Biosciences). The western blots were incubated with 100-fold diluted anti-c-myc antibody followed by secondary antibody conjugated to horseradish peroxidase (GE Healthcare). The myc-TaAGC1 proteins were visualized using the ECL Western Blot Detection and Analysis System (GE Healthcare).

### Analysis of the transcription levels of target genes

Quantitative RT-PCR (qRT-PCR) was used to investigate the relative expression levels of *TaAGC1* and the genes encoding ROS-scavenging enzymes (POX2, CAT1 and SOD3) and ROS-producing enzyme NADPH oxidase (NOX), and four defence-associated genes (*defensin*, *nsLTP1*, *chitinase 2* and *PR10*) in wheat. qRT-PCR was performed using SYBR Green I Master Mix (TaKaRa) in a volume of 25 μl on an ABI 7300 RT-PCR system (Applied Biosystems). Reactions were set up using the following thermal cycling profile: 95°C for 5min, followed by 41 cycles of 95°C for 15 s and 60°C for 31 s. The products were examined using a melting curve analysis program. Three biological replications were run. The relative expression of the target genes was calculated using the 2^−ΔΔCT^ method (Livak and Schmittgen, 2001), where the wheat *Actin* gene, *TaActin*, was used as the internal reference. RT-PCR was used to investigate the transcription of BSMV *CP* gene in VIGS experimental wheat plants.

The accession number of all the genes and sequences of all the primers in the study are listed in Supplementary Table S2.

### Assessment on *R. cerealis* responses

In T_1_−T_3_ generations, 30 plants for each line of the TaAGC1-overexpressing transgenic and WT wheat were inoculated with small toothpicks harbouring the well-developed mycelia of *R. cerealis* following the protocol of Chen *et al.* (2008). The infection types (ITs) of wheat plants and disease index for each line were evaluated at 50 dpi. Based on the disease lesion squares on the base stems (Supplementary Fig. S2), ITs were categorized from 0 to 5: 0, no lesion; 1, the lesion appeared on the sheaths rather than stems; 2, the width of the lesion is <50% of the infected stem perimeter; 3, the width of the lesion is >50 and <75% of the infected stem perimeter; 4, the width of the disease lesion is >75% of the infected stem perimeter; 5, white spike or dead plant). Disease index ={(0×X_0_+1×X_l_+2×X_2_+3×X_3_+4×X_4_+5×X_5_)/[(X_0_+X_1_+X_2_+X_3_+X_4_+X_5_)×5]}×100, where X_0_−X_5_ indicated plants with IT: 0−5.

### Assay of ROS level

*In situ* H_2_O_2_ and O_2_^−^ accumulations were examined via histochemical staining by 3, 3′-diaminobenzidine (DAB) and nitroblue tetrazolium (NBT), respectively, following the protocols of Lee *et al.* (2002). Briefly, leaves were harvested at the indicated time-points after *R. cerealis* infection, and floated in a solution of 1mg ml^−1^ DAB-HCl (pH 3.8) at 25°C for 8h or in NBT (pH 7.8) at 25°C for 30min, and then chlorophyll was removed with 95% ethanol in a boiling-water bath.

## Results

### *TaAGC1* transcription is associated with defence response to *R. cerealis*

**Fig. 1.**
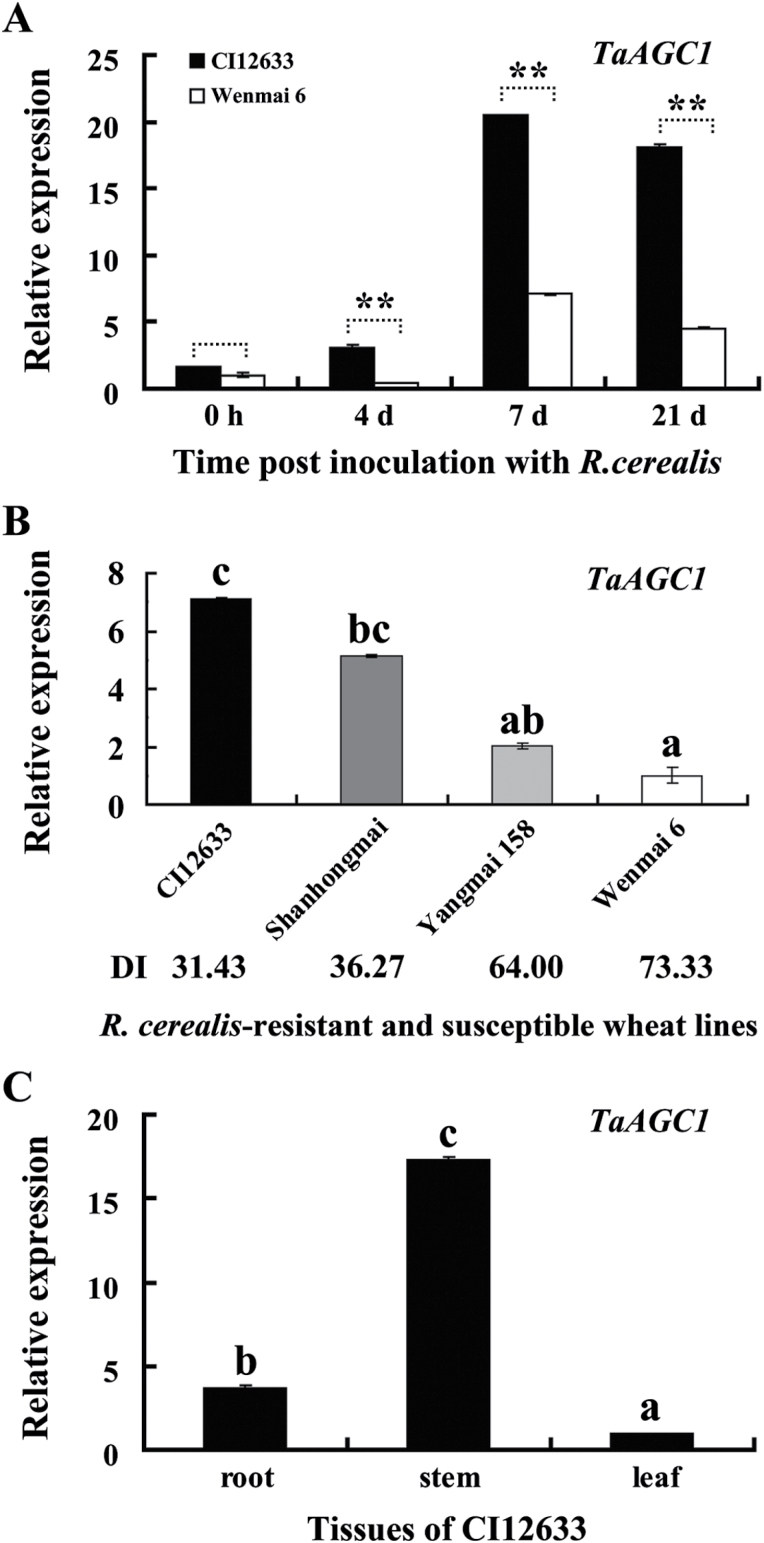
Transcription of the AGC kinase gene *TaAGC1* in *Rhizoctonia cerealis*-inoculated wheat (*Triticum aestivum*) as measured by qRT-PCR. (A) Transcription of *TaAGC1* in stems of the *R. cerealis*-resistant wheat line CI12633 and susceptible wheat cultivar Wenmai 6 at 0h, and 4, 7 and 21 dpi with *R. cerealis*. The expression level of *TaAGC1* in Wenmai 6 plants at 0 dpi is set to 1. The expression levels of *TaAGC1* in CI12633 are relative to those in Wenmai 6 plants at each time point (0h, 4, 7 or 21 dpi). Statistically significant differences between CI12633 and Wenmai 6 at the same time point are derived from the results of three independent replications (*t*-test: **, *P*<0.01). (B) Expression patterns of *TaAGC1* in four wheat cultivars with different degrees of resistance at 21 dpi with *R. cerealis*. DI indicates disease index of sharp eyespot. The expression level of *TaAGC1* in the Wenmai 6 plants was set to 1. (C) Transcription of *TaAGC1* in roots, stems and leaves of the *R. cerealis*-resistant line CI12633 at 21 dpi with *R. cerealis*. The transcriptional levels and standard error are derived from the average of three biological replicates. The transcript levels with different letters are significantly different from each other based on statistical comparisons (*t*-test; *P*<0.01).

To identify kinase genes involved in the defence response to *R. cerealis*, the hybridization data of the Agilent wheat microarray chips were mined for wheat kinase genes that were differentially expressed between sharp eyespot-resistant wheat lines CI12633/Shanhongmai and the susceptible wheat line Wenmai 6 inoculated with *R. cerealis* for 4 and 21 dpi. Among differentially expressed kinase gene sequences identified from microarray data (GEO accession number GSE69245), the transcriptional level of TC411796 (probe), a cDNA 3′-terminal fragment of a wheat AGC kinase, was higher in resistant wheat lines CI12633/Shanhongmai than in susceptible wheat line Wenmai 6. For example, the probe TC411796 showed a 3.01-fold and 2.90-fold transcriptional increase in the resistant wheat line CI12633 than in the susceptible wheat line Wenmai 6 at 4 and 21 dpi, respectively. Being homologous to AGC kinases, this protein kinase gene was designated as *TaAGC1*. qRT-PCR analyses showed that the transcription of *TaAGC1* was induced after *R. cerealis* inoculation, and the transcriptional induction was higher in resistant CI12633 than in susceptible Wenmai 6 at 4, 7 or 21 dpi with *R. cerealis* (Fig. 1A). The qRT-PCR results were in agreement with the microarray analysis trend. Furthermore, the gene transcriptional level was the highest in resistant wheat line CI12633, and the lowest in the highly-susceptible cultivar Wenmai 6 (Fig. 1B). Additionally, the transcript abundance of *TaAGC1* was the highest in stems that are main disease-occurring sites, intermediate in roots, and lowest in the leaves of CI12633 (Fig. 1C). These results suggested that *TaAGC1* transcription might participate in the defence response to *R. cerealis* in resistant wheat lines.

### *TaAGC1* encodes an NDR-type AGC kinase

**Fig. 2.**
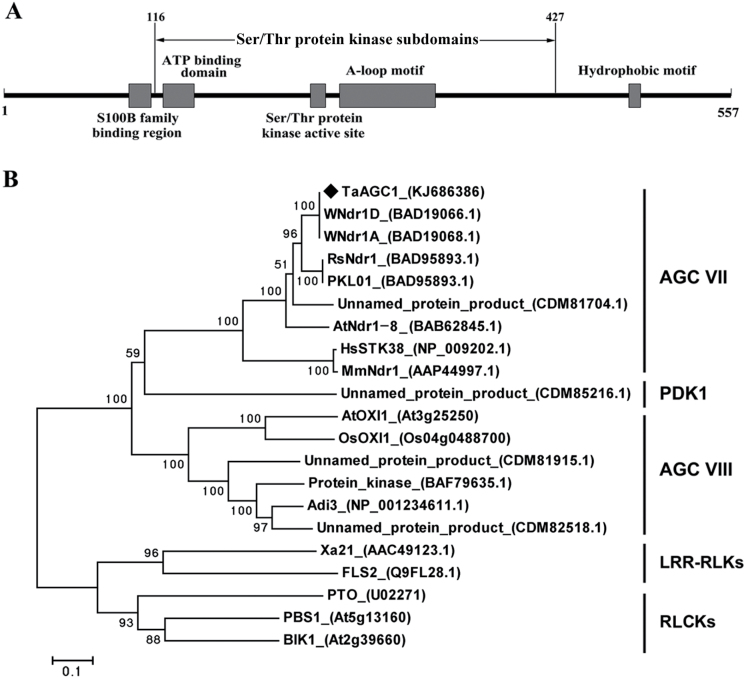
Domain and phylogenetic analyses of the wheat (*Triticum aestivum*) AGC kinase TaAGC1. (A) Schematic diagram of the domain structure of the TaAGC1 protein. Individual domains of TaAGC1 are represented by grey boxes, and the names of motifs are indicated on top or below. (B) Phylogenetic analysis of the deduced amino acid sequences of TaAGC1. The bootstrapped phylogenetic tree is constructed using the neighbor-joining phylogeny of MEGA 5.0. The black diamond indicates the position of TaAGC1. LRR-RLKs indicates leucine-rich repeat receptor-like kinases; RLCKs indicates receptor-like cytoplasmic kinases.

The full-length of *TaAGC1* cDNA sequence (GenBank accession KJ686386) was cloned from a *R. cerealis*-infected stem of CI12633. A sequence analysis showed that the cDNA sequence (2077 nucleotides) of *TaAGC1* includes an ORF consisting of 1674 nucleotides (from 67 to 1740) (Supplementary Fig. S3). The deduced TaAGC1 protein consists of 557 amino acid residues, with a MW of 64.14 kD and pI of 8.65. As shown in Fig. 2A and Supplementary Fig. S3, the TaAGC1 protein contains an N-terminal S100B family-binding region (amino acid 96–113), the 12 Ser/Thr protein kinase subdomains (amino acid 116–427) that harbours an ATP-binding domain (amino acid 122−146), the kinase active-site (amino acid 235−247) and an activation loop (A-loop) motif (amino acid 257−331), and a C-terminal hydrophobic motif (amino acid 479−488).

BLAST and sequence analyses indicated that the TaAGC1 protein sequence was homologous to those of plant Ser/Thr protein kinases and shared the characteristic activation loop signature motif (DFGx_60_STVGTPDYIAPE) of the NDR subclass of the AGC kinase family (Bogre *et al.*, 2003; Supplementary Fig. S3). The reconstructed phylogenetic tree analysis (Fig. 2B) indicated that four wheat AGC kinases including TaAGC1, WNdr1A (GenBank accession no. BAD19068.1), WNdr1D (GenBank accession no. BAD19066.1) and an unnamed wheat AGC protein (GenBank accession no. CDM81704.1), and NDR protein kinases from other species, including the *Arabidopsis thaliana* NDR-type AGC AtNdr-8 (At5g09890, GenBank accession no. BAB62845), radish NDR kinase RsNdr1 (GenBank accession no. BAC76894), *Lotus japonicus* NDR kinase PKL01 (GenBank accession no. BAD95893), human NDR kinase STK38 (GenBank accession no. NP_009202.1) and *Mus musculus* NDR kinase MmNdr1 (GenBank accession no. AAP44997), were clustered on the same branch. All of them belong to AGC VII subfamily. AtOXI1, OsOXI1, Adi3 and three unnamed wheat AGC kinases (GenBank accession nos CDM81915.1, BAF79635.1, and CDM82518.1) were clustered in the other clade, which belonged to the AGC VIIIb subfamily. Another unnamed wheat AGC kinase (GenBank accession no. CDM85216.1) belonged to the PDK1 subfamily. However, the leucine-rich repeat receptor-like kinases, such as rice Xa21 (GenBank accession no. AAC49123.1) and *Arabidopsis* FLS2 (GenBank accession no. Q9FL28.1), and receptor-like cytoplasmic kinases, such as tomato PTO, *Arabidopsis* PBS1 (At5g13160) and BIK1 (At2g39660), were separately clustered into two out-groups, which do not belong to the AGC kinase family (Fig. 2B). The results suggested that the TaAGC1 protein was a member of the NDR-type AGC kinases.

### TaAGC1 possesses autophosphorylation and phosphorylation capability

**Fig. 3.**
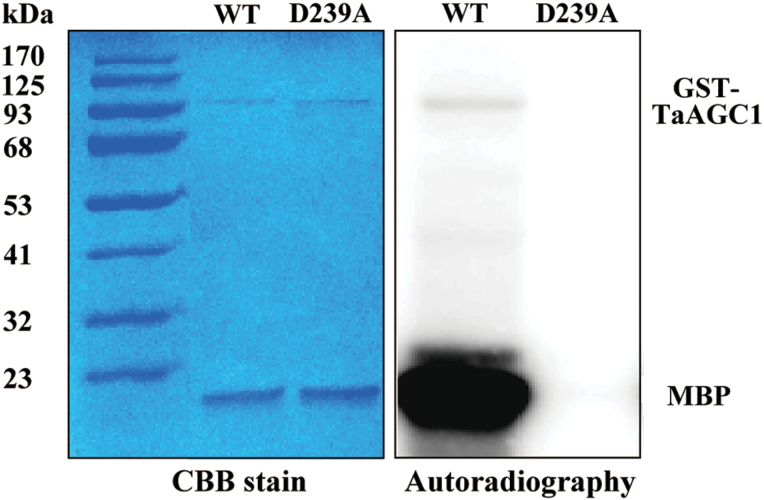
Analysis of the kinase activity of the TaAGC1 protein. The assays on autophosphorylation of TaAGC1 and myelin basic protein (MBP) phosphorylation by TaAGC1 were incubated for 30min in kinase buffer containing purified GST-TaAGC1 (WT) or GST-D239A mutant protein, the MBP substrate and [γ-^32^P] ATP. The phosphorylated proteins were separated by SDS-PAGE and visualized by autoradiography. After autoradiography, the right proteins were confirmed by Coomassie Brilliant Blue (CBB) staining. (This figure is available in colour at *JXB* online.)

To investigate whether TaAGC1 is an active kinase, autophosphorylation and MBP phosphorylation of TaAGC1 were studied. The GST-TaAGC1 (wild-type) fusion protein with 93.2 KD and the GST-D239A (mutant) protein were separately expressed in *E. coli* cells and purified by affinity chromatography. In D239A mutant of TaAGC1, Asp-239 (D) at the Ser/Thr kinase domain active-site was replaced with Ala (A). The *in vitro* kinase assay showed that through detection by autoradiography, TaAGC1 autophosphorylated itself and phosphorylated the substrate MBP, but the D239A mutant did not display the autophosphorylation and phosphorylation activity. These results indicated that TaAGC1 is a functional kinase with autophosphorylation activity and phosphorylation activity, and that the Asp-239 residue of TaAGC1 is required for the TaAGC1 kinase activity (Fig. 3).

### TaAGC1 localizes in the cytoplasm and the nucleus

**Fig. 4.**
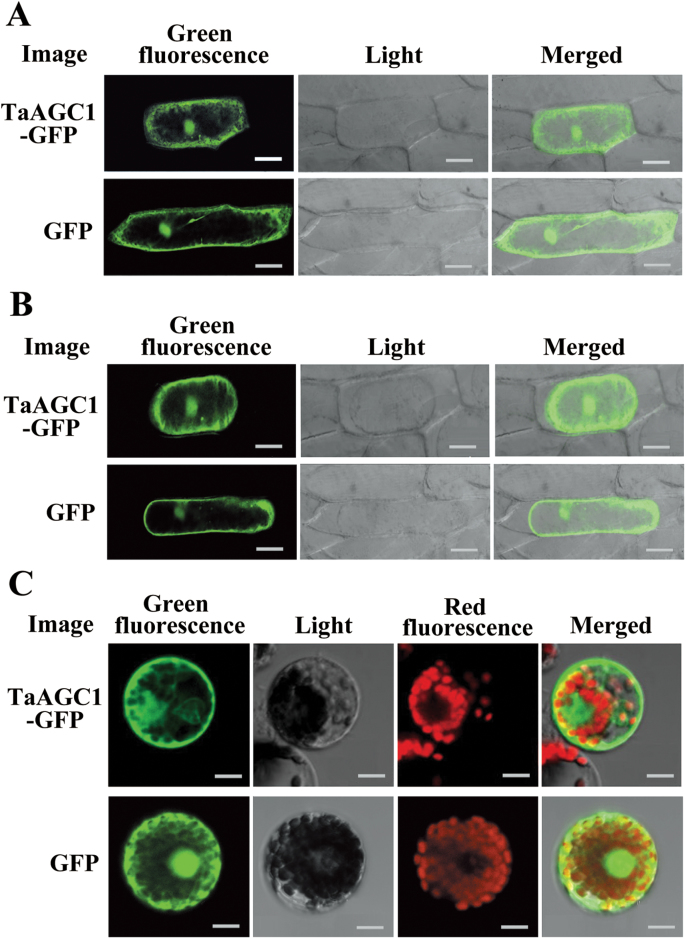
Subcellular localization of the wheat (*Triticum aestivum*) AGC kinase TaAGC1-green fluorescent protein (GFP) fusion protein in onion and wheat. (A) The fused TaAGC1-GFP (upper panel) and control GFP (lower panel) in onion epidermal cells. (B) The fused TaAGC1-GFP (upper panel) and control GFP (lower panel) in plasmolysed onion epidermal cells. Bars, 50 μm (A, B). (C) The fused TaAGC1-GFP (upper panel) and control GFP (lower panel) in wheat protoplasts. Bar, 10 μm. (This figure is available in colour at *JXB* online.)

To explore the subcellular localization of TaAGC1, 35S:TaAGC1-GFP was transiently expressed either in onion epidermal cells or in wheat mesophyll protoplasts. At the same time, the transient expression of 35S:GFP was used as control. In the transient transformed onion epidermal cells, the TaAGC1-GFP protein was localized predominantly in the cytoplasm and nucleus (Fig. 4A). After plasmolysis of the bombarded onion epidermal cells, localization of TaAGC1 was more clearly observed in the cytoplasm and nucleus (Fig. 4B). Furthermore, the green fluorescent signal of the 35S:TaAGC1-GFP protein in wheat mesophyll protoplasts was detected in the cytoplasm and nucleus (Fig. 4C). The green fluorescent signal of the 35S:GFP protein was distributed in the nucleus and cytoplasm (Fig. 4A−C). Thus, the TaAGC1 protein localizes in the cytoplasm and nucleus in wheat.

### Knock-down of *TaAGC1* impairs wheat resistance to *R. cerealis*

**Fig. 5.**
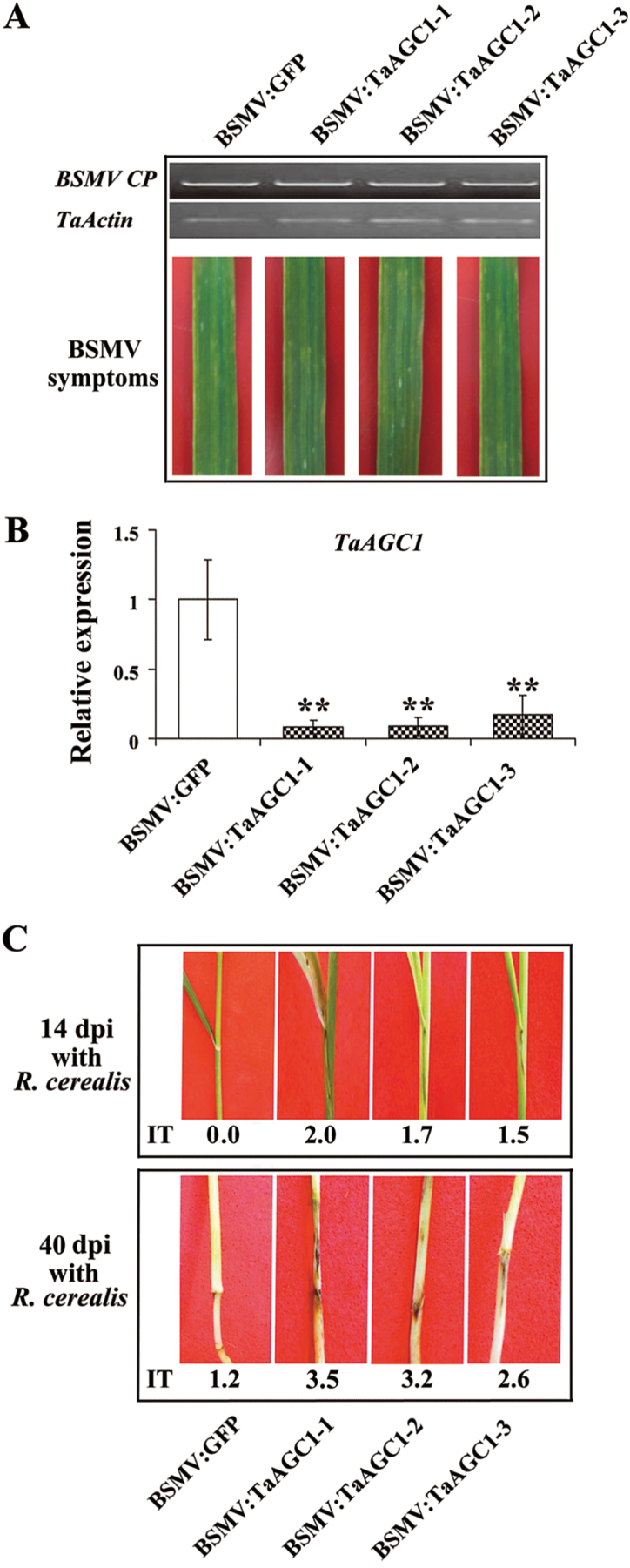
Effect of knock-down of the wheat (*Triticum aestivum*) AGC kinase *TaAGC1* gene on the resistance response of the resistant wheat strain CI12633 to *Rhizoctonia cerealis*. (A) RT-PCR analysis of the transcription levels of the barley stripe mosaic virus (BSMV) *CP* gene and typical symptoms of BSMV in the fourth leaves of wheat plants infected by BSMV:GFP or BSMV:TaAGC1. (B) qRT-PCR analysis of the relative transcript levels of *TaAGC1* in the wheat plants infected by BSMV:GFP or BSMV:TaAGC1 at 14 dpi with *R. cerealis*. The relative transcript level of *TaAGC1* in the wheat CI12633 plants infected by BSMV:TaAGC1 is relative to that in BSMV:GFP-infected wheat CI12633 plants. Three biological replicates per line were averaged and statistically treated (*t*-test; ** *P*<0.01). Bars indicate standard error of the mean. (C) Sharp eyespot symptoms of the BSMV:GFP- and BSMV:TaAGC1-inoculated CI12633 plants at 14 and 40 dpi with *R. cerealis*. Similar results were obtained from three independent replicates. (This figure is available in colour at *JXB* online.)

After the resistant-wheat CI12633 plants had been infected with BSMV for 10 d, symptoms and transcript analyses indicated that BSMV was present in both BSMV:GFP- and BSMV:TaAGC1-infected CI12633 plants (Fig. 5A). The *TaAGC1* transcript level was substantially reduced in BSMV:TaAGC1-infected CI12633 plants, indicating that *TaAGC1* was successfully silenced in BSMV:TaAGC1-infected CI12633 plants (Fig. 5B). Then, these plants were further inoculated with *R. cerealis* to evaluate the defence role of *TaAGC1*. At 14 dpi with *R. cerealis*, a dark brown margin, an early symptom of sharp eyespot, was present on the sheaths of BSMV:TaAGC1-infected CI12633 plants, but absent in BSMV:GFP-treated CI12633 plants. At 40 dpi with *R. cerealis*, obvious symptoms of sharp eyespot were present on the sheaths and stems of BSMV:TaAGC1-infected CI12633 plants, but not on the stems of BSMV:GFP-treated CI12633 plants (Fig. 5C). Compared with BSMV:GFP-treated CI12633 plants, no obvious phenotypic change was observed in BSMV:TaAGC1-infected wheat CI12633 plants except the sharp eyespot resistance alteration. These results indicated that the down-regulation of *TaAGC1* transcripts in CI12633 impaired host resistance to *R. cerealis*, and that *TaAGC1* is required for the wheat defence against *R. cerealis* infection.

### Overexpression of *TaAGC1* enhances wheat resistance to sharp eyespot

**Fig. 6.**
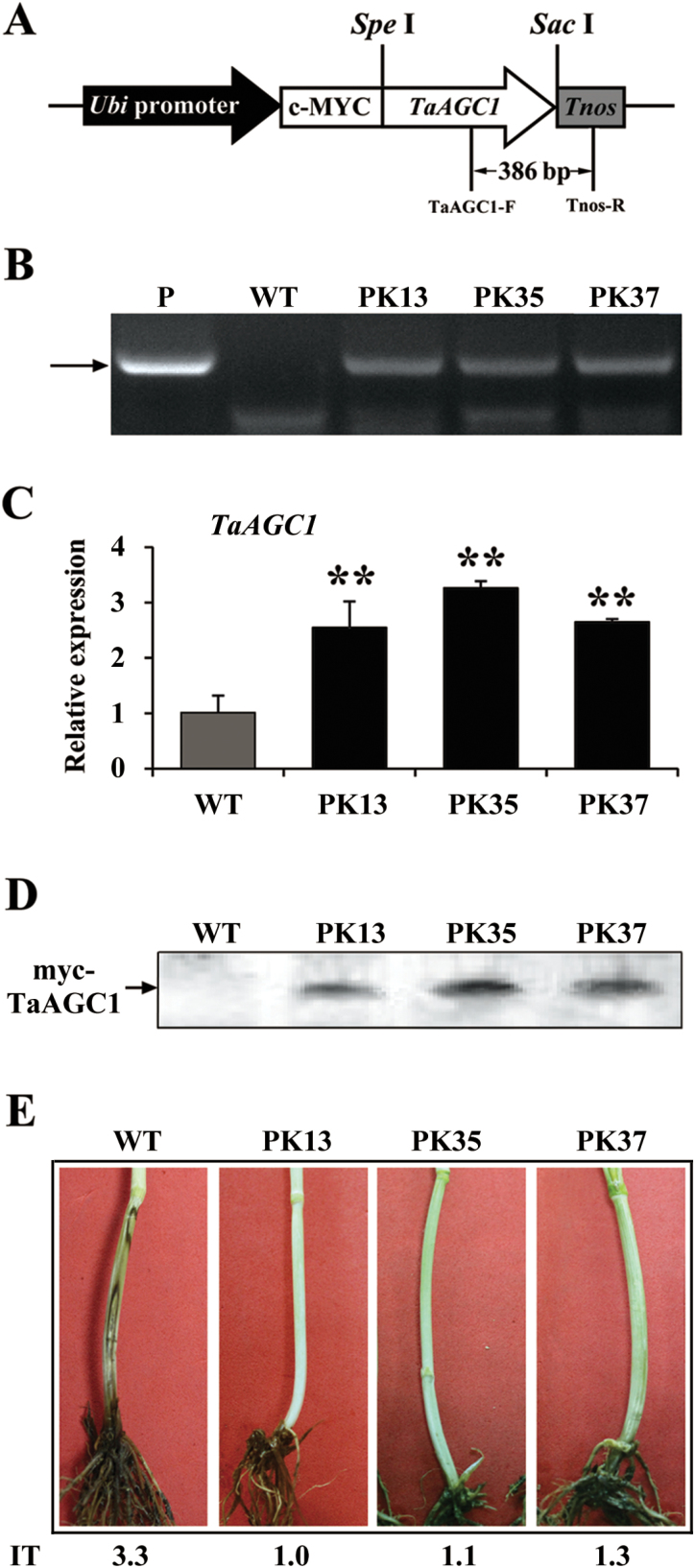
Molecular analyses and *Rhizoctonia cerealis* responses of the wheat (*Triticum aestivum*) AGC kinase gene *TaAGC1*-overexpressing transgenic wheat plants. (A) *TaAGC1*-overexpressing transformation vector pUBI:myc-TaAGC1. The arrow indicates the fragment amplified in the PCR detection of the transgene. (B) PCR pattern of three *TaAGC1*-overexpressing transgenic lines (PK13, PK35 and PK37) and wild-type (WT) wheat Yangmai 20 using the *TaAGC1*-overexpressing transgene specific primers. P, the transformation vector pUBI:myc-TaAGC1 as a positive control. (C) qRT-PCR analyses of the relative transcript levels of TaAGC1 in three TaAGC1 transgenic lines at 7 d post *R. cerealis* inoculation. The relative transcript level of *TaAGC1* in transgenic lines is relative to that in the WT plants. Three biological replicates per line were averaged and statistically treated (*t*-test; ** *P*<0.01). Bars indicate standard error of the mean. (D) Western blot pattern of the three *TaAGC1*-overexpressing transgenic lines and WT Yangmai 20 using an anti-myc antibody. Similar results were obtained from three independent replicates. (E) Typical symptoms of sharp eyespot in the three *TaAGC1*-overexpressing transgenic and WT Yangmai 20 plants. IT indicates infection type. (This figure is available in colour at *JXB* online.)

The role of *TaAGC1* in the resistance to *R. cerealis* was further studied by developing and evaluating *TaAGC1*-overexpressing transgenic wheat lines. PCR and qRT-PCR assays showed that the transcript abundance of *TaAGC1* in three stably-overexpressing transgenic lines (PK13, PK35 and PK37) was markedly elevated compared with untransformed recipient (wild type, WT) Yangmai 20 (Fig. 6B, C). Western blotting indicated that the introduced *myc-TaAGC1* gene was translated into the fusion protein in the three *TaAGC1*-overexpressing transgenic lines, but not in WT Yangmai 20 (Fig. 6D). The sharp eyespot severity assessments in three successive (T_1_−T_3_) generations showed that, compared with WT Yangmai 20, all three *TaAGC1*-overexpressing lines (PK13, PK35 and PK37) displayed significantly enhanced resistance to *R. cerealis* infection (Table 1, Fig. 6E). For example, the average ITs and disease indices of the three *TaAGC1*-overexpressing wheat lines in T_2_ generation were 1.79–2.16 and 37.14–43.43%, respectively, whereas the average IT and disease index of the WT Yangmai 20 were 3.60 and 76.89%, respectively (Table 1). These results proved that *TaAGC1* positively regulated wheat defence responses to *R. cerealis* infection.

**Table 1.** *Rhizoctonia cerealis* responses in transgenic wheat (*Triticum aestivum*) lines overexpressing the AGC kinase gene *TaAGC1* and wild-type (WT) wheat

**Lines**	**Infection type**			**Disease index**		
	**T**_**1**_	**T**_**2**_	**T**_**3**_	**T**_**1**_	**T**_**2**_	**T**_**3**_
PK13	1.38**	2.17**	1.63**	27.50**	43.43**	32.66**
PK35	1.63**	1.86**	1.62**	32.50**	37.14**	32.36**
PK37	1.62**	1.98**	1.73**	32.42**	39.67**	34.61**
WT (Yangmai 20)	2.28	3.84	2.72	45.56	76.89	54.38

** Significant difference between each transgenic line and untransformed (WT) wheat Yangmai 20 at *P*<0.01 (*t*-test). Infection types (ITs) were categorized from 0 to 5: 0, no lesion; 1, the lesion appeared on the sheaths rather than stems; 2, the width of the lesion is <50% of the infected stem perimeter; 3, the width of the lesion is >50 and <75% of the infected stem perimeter; 4, the width of the disease lesion is >75% of the infected stem perimeter; 5, white spike or dead plant. The infection types were the average ITs of 30 plants for each line of the *TaAGC1* transgenic wheat in T_1_−T_3_ generations and WT wheat Yangmai 20 at 50 dpi with *R. cerealis*. Disease index={(0×X_0_+1×X_l_+2×X_2_+3×X_3_+ 4×X_4_+5×X_5_)/[(X_0_+X_1_+X_2_+X_3_+X_4_+X_5_)×5]} ×100, where X_0_−X_5_ indicated plants with IT: 0−5. The representative symptoms of different ITs are shown in Supplementary Fig. S2.

### *TaAGC1* modulates ROS and related gene expression levels

**Fig. 7.**
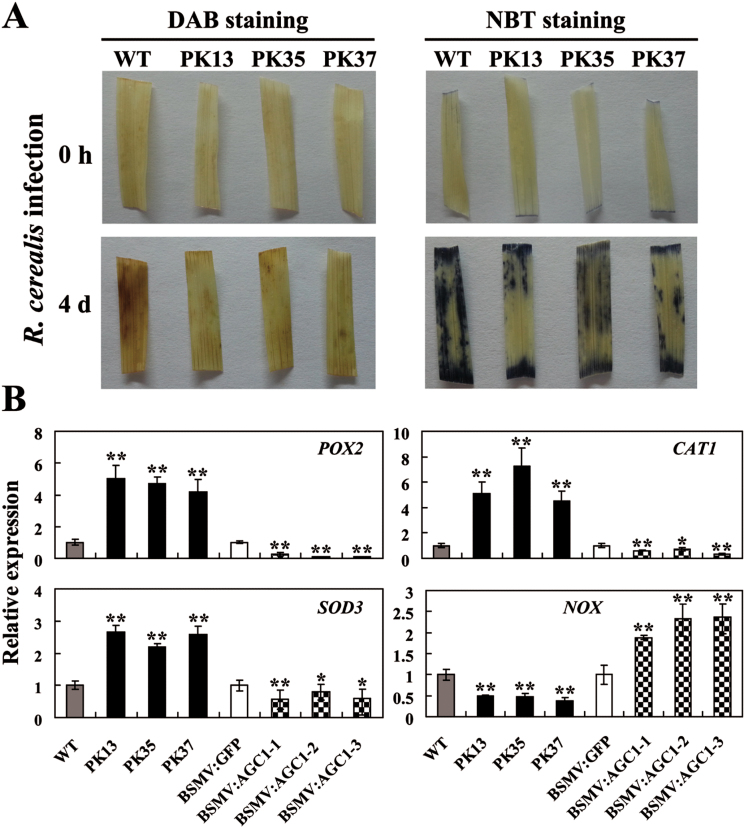
Wheat (*Triticum aestivum*) AGC kinase TaAGC1 modulates reactive oxygen species (ROS) levels and the expression of ROS-related genes. (A) Analysis of hydrogen peroxide (H_2_O_2_) and superoxide anion (O_2_^−^) levels in wheat. Leaves were harvested from *TaAGC1*-overexpressors (PK13, PK35 and PK37) and WT wheat plants at 0h and 4 dpi with *Rhizoctonia cerealis*, and were then stained with 3,3ʹ-diaminobenzidine (DAB) and nitroblue tetrazolium (NBT) to detect H_2_O_2_ and O_2_^−^, respectively. Similar results were obtained from three independent replicates. (B) Transcriptional analysis of genes encoding ROS-scavenging enzymes (POX2, CAT1 and SOD3) and the ROS-producing enzyme NOX in wheat. The tested wheat samples include *TaAGC1*-overexpressors (PK13, PK35 and PK37), untransformed wheat Yangmai 20 (WT) and *TaAGC1*-knock-down plants (BSMV:TaAGC1-1, -2 and -3) and BSMV-GFP-infected controls at 7 dpi with *Rhizoctonia cerealis*. The reported transcript levels of the tested gene in the transgenic plants are relative to those in the WT plants. Statistically significant differences of *TaAGC1*-overexpression or *TaAGC1*-knock-down wheat plants were compared with the WT or the control, and based on three technical replications (*t*-test; *P<0.05, **P<0.01). Bars indicate standard error of the mean. (This figure is available in colour at *JXB* online.)

The expression of *TaAGC1* was significantly induced after the H_2_O_2_ treatment from 1h to 12h and reached a peak at 3h post-treatment (Supplementary Fig. S4), suggesting that *TaAGC1* might be involved in ROS signalling. To explore if the accumulation of ROS occurs in wheat challenged by *R. cerealis*, levels of H_2_O_2_ and O_2_^−^ in the infected-wheat Yangmai 20 were assayed using DAB and NBT staining, respectively. The results showed that levels of H_2_O_2_ and O_2_^−^ increased in the infected-wheat leaves from 1 dpi to 7 dpi (Fig. 7A, Supplementary Fig. S5), suggesting that the *R. cerealis* infection promoted the generation of H_2_O_2_ and O_2_^−^. To unravel whether *TaAGC1* regulates ROS homeostasis, levels of H_2_O_2_ and O_2_^−^ in *TaAGC1*-overexpressing and WT Yangmai 20 leaves after *R. cerealis* infection were further assayed. Under normal growth conditions, either WT or *TaAGC1*-overexpressing plants produced a small amount of H_2_O_2_ and O_2_^−^ (Fig. 7A). Following *R. cerealis* infection, there were less DAB- and NBT-stained spots on the leaves of *TaAGC1*-overexpressing plants than on WT plants (Fig. 7A), suggesting that the *TaAGC1* overexpression reduced the accumulation of H_2_O_2_ and O_2_^−^ in the transgenic wheat.

To explore if *TaAGC1* modulated ROS-related genes, the transcription of wheat genes encoding ROS-scavenging enzymes (POX2, CAT1 and SOD3) and ROS-producing enzyme (NOX) in *TaAGC1*-overexpressing, *TaAGC1*-knock-down and control wheat plants were investigated. Under normal conditions, the transcriptional levels of *POX2*, *CAT1* and *SOD3* genes were significantly higher but the *NOX* transcript level was significantly lower in *TaAGC1*-overexpressing lines than in WT plants (Supplementary Fig. S6), suggesting that *TaAGC1* positively regulated the transcription of *POX2*, *CAT1* and *SOD3* genes but negatively regulated the expression of *NOX*. Furthermore, following *R. cerealis* inoculation for 7 d and 40 d, the induced expression levels of *POX2*, *CAT1* and *SOD3* genes were significantly higher in *TaAGC1* overexpressing wheat lines than in the WT plants and were lowest in the *TaAGC1* knock-down plants. However, the *NOX* transcript level was significantly lower in *TaAGC1*-overexpressing lines than in WT plants and was the highest in *TaAGC1*-knock-down plants (Fig. 7B; Supplementary Fig. S7). The data suggested that the overexpression of *TaAGC1* increased the expression of *POX2*, *CAT1* and *SOD3*, but decreased the expression of *NOX*, resulting in a reduced ROS content in the overexpressing transgenic wheat plants infected by *R. cerealis*.

### *TaAGC1* regulates the expression of defence-associated genes

**Fig. 8.**
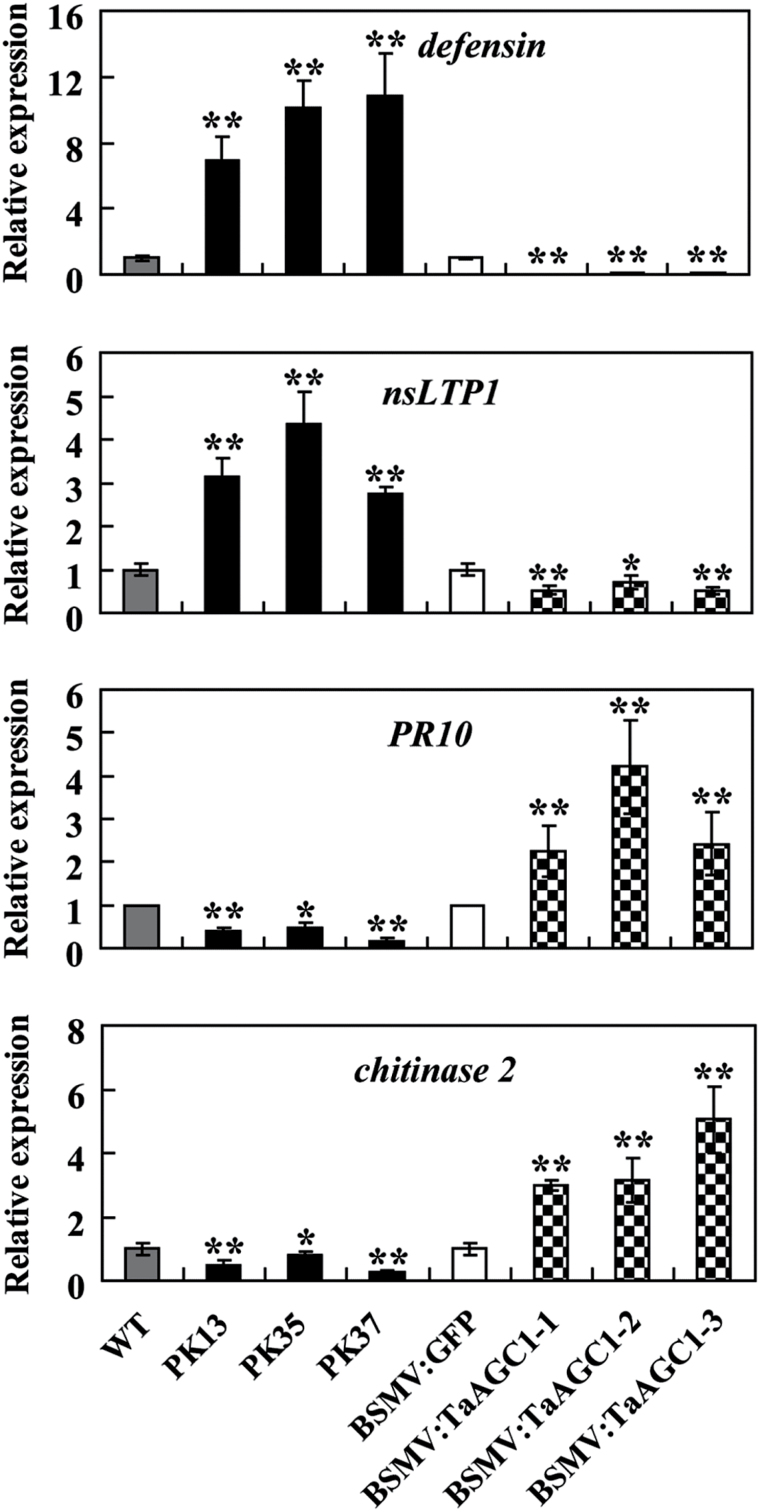
Transcriptional analysis of four defence marker genes (*defensin*, *nsLTP1*, *chitinase 2* and *PR10*) in wheat (*Triticum aestivum*). The tested wheat samples include *TaAGC1*-overexpressing lines (PK13, PK35 and PK37), untransformed Yangmai 20 (WT) and *TaAGC1*-knock-down plants (BSMV:TaAGC1-1, -2 and -3) and BSMV-GFP- infected controls at 7 dpi with *Rhizoctonia cerealis*. The reported transcript levels of the tested genes in the transgenic plants are relative to those in the WT plants. Statistically significant differences of *TaAGC1*-overexpression or *TaAGC1*-knock-down wheat plants were compared with WT or the controls, and based on three technical replications (*t*-test; *P<0.05, **P<0.01). Bars indicate standard error of the mean.

qRT-PCR analysis showed that the expression of *TaAGC1* and four wheat defence-associated genes, *defensin*, *nsLTP1*, *PR10* and *chitinase 2*, was significantly induced in WT wheat Yangmai 20 after *R. cerealis* inoculation for 7 d, 14 d or 40 d (Supplementary Fig. S8). To explore if *TaAGC1* regulates the expression of these defence-associated genes in wheat, qRT-PCR was used to examine their transcription in *TaAGC1*-overexpressing, *TaAGC1*-knock-down, and control wheat plants. Under normal conditions, the transcriptional levels of wheat *defensin* and *nsLTP1* genes were significantly higher but those of *PR10* and *chitinase 2* were significantly lower in *TaAGC1*-overexpressing lines than in WT plants (Supplementary Fig. S6). Furthermore, following *R. cerealis* inoculation for 7 d and 40 d, the transcriptional levels of wheat *defensin* and *nsLTP1* genes elevated significantly more in *TaAGC1*-overexpressing lines than in WT plants and were the lowest in the *TaAGC1*-knock-down lines (Fig. 8; Supplementary Fig. S7). However, the transcriptional levels of *PR10* and *chitinase 2* were decreased in *TaAGC1*-overexpressing resistant lines than in WT plants and were the highest in the *TaAGC1*-knock-down plants (Fig. 8; Supplementary Fig. S7). The data suggested that *TaAGC1* positively regulated the expression of wheat *defensin* and *nsLTP1* genes but negatively regulated the expression of *chitinase 2* and *PR10*.

## Discussion

In this study, an NDR-type AGC gene in wheat, *TaAGC1*, was isolated based on the microarray analysis and the qRT-PCR analyses of transcription levels in resistant and susceptible wheat genotypes. It was expressed at a higher level in *R. cerealis*-resistant wheat line CI12633 than in susceptible wheat line Wenmai 6. To understand the relationship between the gene transcriptional variance and the sequences in the resistant and susceptible wheat genotypes, we cloned the *TaAGC1* sequence from the *R. cerealis*-resistant wheat line CI12633 and the homologous sequence from *R. cerealis*-susceptible wheat line Wenmai 6, respectively. Comparison of *TaAGC1* sequences from CI12633 and Wenmai 6 showed that four single-nucleotide polymorphisms existed at their 3ʹ-terminal sequences (Supplementary Fig. S9), which may be a reason for the different expression patterns in the resistant and susceptible wheat genotypes. The protein TaAGC1 was predicted to be a Ser/Thr protein kinase and it shows the sequence characteristics (like the activation loop signature motif, DFGx_60_STVGTPDYIAPE) of NDR-type AGC kinases. For human NDR-type AGC kinases, three regulatory phosphorylation sites, Thr-74 in the S100B-binding domain, Ser-281 in the catalytic domain and Thr-444 in the hydrophobic motif, were previously reported to be crucial for NDR1 activity (Tamaskovic *et al.*, 2003). All three phosphorylation sites are also found in the TaAGC1 sequence (Supplementary Fig. S3). A phylogenetic analysis provided additional evidence that the TaAGC1 protein is an NDR-type AGC kinase and is distinct from receptor-like kinases or receptor-like cytoplasmic kinases. The majority of receptor-like kinases contain transmembrane and extracellular domains and localize to the plasma membrane, which is consistent with their function in sensing extracellular signals (AbuQamar *et al.*, 2008). The TaAGC1 protein lacks transmembrane and extracellular domains. Computational tools, such as WoLF PSORT (Horton *et al.*, 2007), Cell-Ploc (Chou and Shen, 2010) and SCLPred (Mooney *et al.*, 2011), predicted that TaAGC1 is likely localized to the cytoplasm and nucleus. Based on fluorescent signals of TaAGC1-GFP or GFP in the transiently transformed wheat and onion cells, TaAGC1 is localized in the cytoplasm and the nucleus in onion epidermal cell and wheat protoplast, which is consistent with the computational prediction. The protein blot analysis could help to further study TaAGC1 subcellular localization in the future. Kinase activity assays confirmed that TaAGC1 protein is a functional kinase with autophosphorylation activity and phosphorylation activity and that the residue Asp-239 (D) at the Ser/Thr kinase active-site is required for the kinase activity of TaAGC1. The biochemical properties are in agreement with the sequence traits.

Many genes are induced by environmental stresses and they offer the potential for improving biotic and abiotic stress tolerance *in planta.* For example, the expression of *TPK1b*, a tomato protein kinase gene, is induced by the necrotrophic fungus *B. cinerea* and wounding stimuli. Accordingly, TPK1b mediates plant responses to *B. cinerea* and insect herbivory (AbuQamar *et al.*, 2008). The expression of the rice AGC kinase gene *OsOxi1* was induced after the infection of the blast fungal pathogen *M. oryzae* pv. *oryzae*, and the *OsOxi1*-overexpression rice plants displayed enhanced resistance to blast disease (Matsui *et al.*, 2010). However, this was not necessarily true in defence responses of distinct plant species against different lifestyle pathogens. For example, in *Arabidopsis*, although the expression of *AtOXI1* was strongly induced after infection with *B. cinerea*, the *Atoxi1* null mutant did not display altered susceptibility to this necrotrophic pathogen (Petersen *et al.*, 2009). Additionally, although *AtOXI1* expression was induced by *H. arabidopsidis* and *P. syringae* pv. *tomato*, both *oxi1* null mutant and *AtOXI1* overexpression lines showed increased susceptibility to virulent *H. arabidopsidis* and to both virulent and avirulent *P. syringae* pv. *tomato*. These data suggest that *OXI1* is required for plant immunity to *H. arabidopsidis* and *P. syringae* pv. *tomato*, and *OXI1* expression levels appear to be critical in mounting an appropriate defence response (Petersen *et al.*, 2009). Here, the expression of *TaAGC1* was significantly induced after *R. cerealis* infection, and the induction was higher in *R. cerealis*-resistant lines than in susceptible lines. Accordingly, following inoculation with *R. cerealis*, *TaAGC1*-overexpressing transgenic wheat lines (PK13, PK35 and PK37) in the T_1_−T_3_ generations displayed significantly enhanced resistance to sharp eyespot disease compared to untransformed recipient plants, while the down-regulation of *TaAGC1* in resistant CI12633 significantly impaired the host resistance. These data indicated that the wheat AGC kinase TaAGC1 positively contributes to host resistance to the necrotrophic fungus *R. cerealis*. Interestingly, compared with host wheat plants, the *TaAGC1*-overexpressing and knock-down wheat plants either did not show obvious phenotypic changes in development or did not alter resistance to powdery mildew disease (Supplementary Fig. S10). It would be interesting to further study how *TaAGC1*-overexpressing and knock-down wheat plants perform in response to other wheat pathogens in the future. In previous studies, three AGC VIIIb kinases in other plant species were reported to participate in the immune responses to biotrophic and hemibiotrophic pathogens, including the *Arabidopsis* AtOXI1 to *H. arabidopsidis* and *P. syringae* pv. *tomato* (Rentel *et al.*, 2004; Petersen *et al.*, 2009), the rice OsOxi1 to *Xanthomonas oryzae* pv. *oryzae* (Matsui *et al.*, 2010), and tomato Adi3 to *P. syringae* pv. *tomato* (Devarenne *et al.*, 2006). The TaAGC1 protein fell into the AGC VII subfamily (namely an NDR-type AGC protein kinase), but was not in the clade containing AtOXI1, OsOXI1 and Adi3. To our knowledge, this is the first report of a defence role for a plant NDR-type AGC kinase, and the first functional characterization of a wheat AGC gene in the resistance response to necrotrophic pathogens. To date, the mechanisms of plant responses to necrotrophic pathogens have been poorly understood. These results extend the current knowledge of defence mechanisms against pathogen attacks in plants.

In early responses to pathogen invasion, plants often generate an excessive amount of ROS. During plant immunity against biotrophic pathogens, ROS not only functions in toxicity and the oxidative cross-linking of plant cell walls, but also has a signalling role in mounting a defence response (Petersen *et al.*, 2009). A kinase cascade and downstream transcription factors represent key regulatory components of the ROS signal transduction cascade (Rentel *et al.*, 2004; Matsui *et al.*, 2010; Schmidt *et al.*, 2013). For example, the *Arabidopsis* AGC kinase AtOxi1 is an essential part of the ROS signal transduction pathway and is required for root hair development and immunity against *H. parastica* and *P. syringae* pv. *tomato*, two separate H_2_O_2_-mediated processes (Rentel *et al.*, 2004; Petersen *et al.*, 2009). Additionally, ROS-scavenging enzymes and antioxidants play significant roles in plant defences against certain necrotrophic pathogens (Kuźniak and Skłodowska, 2004; Sharma *et al.*, 2007). In this study, in wheat response to the infection of the necrotrophic pathogen *R. cerealis*, a signalling cascade in wheat is triggered, which includes the activation of *TaAGC1* and the accumulation of ROS. TaAGC1 is responsive to H_2_O_2_ stimuli. Importantly, the expression of the ROS-scavenging enzyme genes (*POX2*, *CAT1* and *SOD3*) is increased in uninfected wheat plants overexpressing *TaAGC1*, suggesting that *TaAGC1* is involved in ROS signalling. Furthermore, after *R. cerealis* infection, the transcription levels of *TaAGC1*, *SOD3*, *POX2* and *CAT1* genes were more elevated in the *TaAGC1*-overexpressing wheat lines compared with the WT line, and were lower in *TaAGC1* knock-down wheat plants than in the control plants, further indicating that TaAGC1 positively regulated the transcription of these ROS-scavenging enzyme genes but down-regulated the expression of the gene *NOX* encoding ROS-producing enzyme. Consequently, following *R. cerealis* infection, the ROS level was lower in *TaAGC1*-overexpressing wheat lines compared with the WT line. These data suggested that *TaAGC1* maintained ROS homeostasis by regulating the expression of ROS-scavenging and -producing enzyme genes.

In plants, defence genes play vital roles in defence responses to pathogens. For example, transgenic wheat plants overexpressing *TaLTP5* (a wheat *LTP* gene) displayed enhanced resistance to the fungal pathogens *Cochliobolus sativus* and *Fusarium graminearum* (Zhu *et al.*, 2012). Overexpression of certain ERF or MYB transcription factors improved wheat resistance to soil-born fungal diseases through up-regulation of wheat defence marker genes (Chen *et al.*, 2008; Zhang *et al.*, 2012; Liu *et al.*, 2013; Zhu *et al.*, 2014). However, different regulatory factors, such as protein kinases and transcription factors, may regulate distinct defence-related genes in plant defence responses to pathogens. In this study, to explore if the wheat AGC kinase TaAGC1 regulates the expression of certain wheat defence-associated genes in TaAGC1-mediated resistance to *R. cerealis*, qRT-PCR was used to investigate expression in four of them (*defensin*, *PR10*, *chitinase 2*, and *nsLTP1*) in *TaAGC1*-overexpressing and knock-down wheat plants, and control wheat plants with or without *R. cerealis* inoculation. These four wheat defence-related genes were reported to participate in transcription factor-mediated defence responses to pathogens in previous studies (Liu *et al.* 2013; Zhu *et al.*, 2014). qRT-PCR analysis results showed that *TaAGC1*, *defensin* and *nsLTP1* genes were up-regulated in the resistant wheat plants overexpressing *TaAGC1* but down-regulated in *TaAGC1*-knock-down susceptible wheat plants following *R. cerealis* challenge. The data suggest that *TaAGC1* positively regulates the expression of *defensin* and *nsLTP1* and that *defensin* and *nsLTP1* might positively contribute to the *TaAGC1*-mediated resistance to *R. cereali*s infection. In contrast, *PR10* and *chitinase 2* genes were down-regulated in the resistant *TaAGC1*-overexpressing wheat plants but up-regulated in susceptible *TaAGC1*-knock-down wheat plants following *R. cerealis* inoculation, suggesting that *TaAGC1* might negatively regulate the expression of *PR10* and *chitinase 2* genes, and that *PR10* and *chitinase 2* might negatively contribute to the *TaAGC1*-mediated resistance. A recent study showed that in pepper, the expression of *PR10* triggered hypersensitive cell death that represses infection of biotrophic pathogens but might facilitate the growth and colonization of necrotrophic pathogens in plants (Choi *et al.*, 2012), supporting our results about *PR10* in wheat. The deep molecular mechanisms underlying TaAGC1 roles remain to be further studied.

In summary, the wheat NDR-type AGC kinase TaAGC1, acting as a positive regulator, mediates host resistance to *R. cerealis* through modulating the expression of ROS-related and certain defence-associated genes. *TaAGC1* can be a candidate gene for improving sharp eyespot resistance in wheat. This study provides novel insights into the defence roles of plant NDR-type AGC kinases against necrotrophic pathogens.

## Supplementary data

Supplementary data is available at *JXB* online.

Supplementary Fig. S1. Scheme of genomic RNAs of the barley stripe mosaic virus (BSMV) construct, BSMV with green fluorescent protein fusion construct (BSMV:GFP), and the construct of the recombinant virus expressing the wheat AGC gene *TaAGC1*, BSMV:TaAGC1.

Supplementary Fig. S2. The representative symptoms of different infection types of sharp eyespot disease at 40 dpi with *Rhizoctonia cerealis.*

Supplementary Fig. S3. Nucleotide sequence and deduced amino acid sequence of the wheat AGC kinase gene *TaAGC1*.

Supplementary Fig. S4. Expression patterns of the wheat AGC kinase gene *TaAGC1* in wheat responding to exogenous hydrogen peroxide (H_2_O_2_) treatments.

Supplementary Fig. S5. Analysis of hydrogen peroxide (H_2_O_2_) and superoxide anion (O_2_^−^) accumulation in wheat leaves.

Supplementary Fig. S6. Transcriptional analysis of ROS-related and defence genes in the wheat AGC kinase gene *TaAGC1*-overexpressing and untransformed wheat Yangmai 20 plants under normal growth conditions.

Supplementary Fig. S7. Transcription analysis of ROS-related and defence genes in the wheat AGC kinase gene *TaAGC1*-overexpressing and knock-down, as well as control wheat plants with *Rhizoctonia cerealis* inoculation for 40 d.

Supplementary Fig. S8. Transcription analysis of ROS-related and defence genes in the wild type wheat Yangmai 20 plants after *Rhizoctonia cerealis* inoculation for 7, 14 and 40 d.

Supplementary Fig. S9. Alignment of 3ʹ terminal sequences of *TaAGC1* in resistant wheat line CI12633 and of its homologue in susceptible wheat line Wenmai 6.

Supplementary Fig. S10. The powdery mildew symptoms of the wheat AGC kinase gene *TaAGC1*-overexpressing and knock-down, and control wheat plants.

Supplementary Table S1. Rhizoctonia cerealis responses of four wheat lines.

Supplementary Table S2. Primers used in this study.

Supplementary Data
