# Supplementary material for: The wheat AGC kinase TaAGC1 is a positive contributor to host resistance to the necrotrophic pathogen Rhizoctonia cerealis
Source: J Exp Bot. 2015 Jul 27;66(21):6591–603. doi: 10.1093/jxb/erv367 (PMC4623678; doi:10.1093/jxb/erv367)
Supplement: Supplementary Data [file supp_66_21_6591__index.html]

The wheat AGC kinase TaAGC1 is a positive contributor to host resistance to the necrotrophic pathogen Rhizoctonia cerealis — The wheat AGC kinase TaAGC1 is a positive contributor to host resistance to the necrotrophic pathogen Rhizoctonia cerealis — Supplementary Data 

# The wheat AGC kinase TaAGC1 is a positive contributor to host resistance to the necrotrophic pathogen *Rhizoctonia cerealis*

## Supplementary Data

Data files

- Supplementary Data - Supplementary Data
