# Supplementary material for: The wheat AGC kinase TaAGC1 is a positive contributor to host resistance to the necrotrophic pathogen Rhizoctonia cerealis
Source: J Exp Bot. 2015 Jul 27;66(21):6591–603. doi: 10.1093/jxb/erv367 (PMC4623678; doi:10.1093/jxb/erv367)
Supplement: Supplementary Data [file supp_erv367_jexbot151431_file001.pdf]

Supplementary data

The wheat AGC kinase TaAGC1 is a positive contributor to host resistance to the necrotrophic pathogen *Rhizoctonia cerealis*

Xiuliang Zhu, Kun Yang, Xuening Wei, Qiaofeng Zhang, Wei Rong, Lipu Du, Xingguo Ye, Lin Qi, Zengyan Zhang

Supplementary Figures

Supplementary Fig. S1 Scheme of genomic RNAs of the barley stripe mosaic virus (BSMV) construct, BSMV with green fluorescent protein fusion construct (BSMV:GFP), and the construct of the recombinant virus expressing the wheat (*Triticum aestivum*) AGC gene *TaAGC1*, BSMV:TaAGC1. The orientation of the *GFP* or *TaAGC1* inserts is indicated by dark boxes.

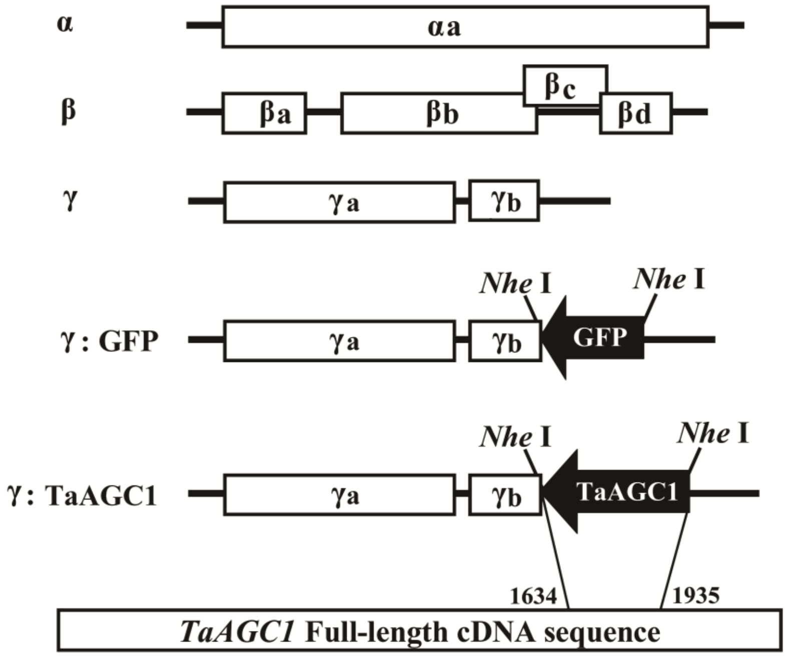

Supplementary Fig. S2 The representive symptoms of different infection types of sharp eyespot disease at 40 d post inoculation with *Rhizoctonia cerealis*. ITs indicates infection types. ITs were categorized from 0 to 5 (“IT:0” = no lesion, “IT:1” = the lesion appeared on the sheaths rather than stems, “IT:2” = the width of the lesion < 50% of the infected stem perimeter, “IT:3” = the width of the lesion >50 and < 75% of the infected stem perimeter, “IT: 4”= the width of the disease lesion more than 75% of the infected stem perimeter, “IT: 5”= white spike or dead plant).

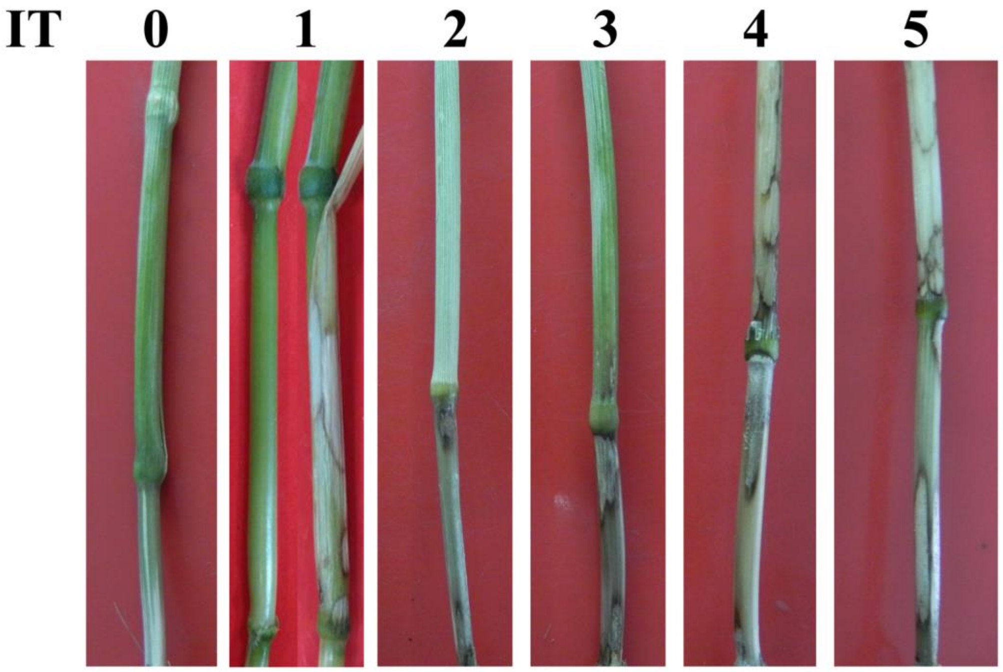

Supplementary Fig. S3 Nucleotide sequence and deduced amino acid sequence of the wheat (*Triticum aestivum*) AGC kinase gene *TaAGC1*. The kinase domain is represented by a single line. Roman numerals mark the subdomains conserved in the plant serine/threonine protein kinase family. Arrowheads indicate the three kinase catalytic sites. The S100B binding region is marked by the double line, the catalytic domain is indicated by the open box, and the C-terminal hydrophobic motif is represented by the wavy line. The long insertion sequence between subdomains VII and VIII is shown by a broken underline. The three phosphorylation sites are shaded in gray.

1 CGGCTGCTGGATTGGTCAATATAATACGCTCTCTGGTCTTGGAGATGATGCCATTGTGAGCACTAGATGGATTCCGCGAGAAGTTGGTTC

1 M D S A R S W F

91 CAGAAGTTCCAGCCGCGGGACAAGTCCAAGAGCCCGGCGGTGCCTGCCAGCCATGGGAAGGATCCCGGGAAGCCCCCCGTCGACGACGCG

9 Q K F Q P R D K S K S P A V P A S H G K D P G K P P V D D A

181 CCTTCTAGCGCGACCAAGCAGAAGGTCGCTGCGGCAAAACAGTACATTGAGAACCACTACAAGACTCAGATGAAGTCCTTGCAAGATAGG

39 P S S A T K Q K V A A A K Q Y I E N H Y K T Q M K S L Q D R

271 AAAGAGAGGCGCTGGATGCTGGAGAGGAAATTACAGGATGCTGAAGTTCCTGCAGAAGAGCAGAACAACATTCTAAAACATTTGGAGAAA

69 K E R R W M L E R K L Q D A E V P A E E Q N N I L K H L E K

361 AAGGAGACTGAATATATGCGTTTGCAAAGACACAAGATGGGGGTTGAAGATTTTGAACTTTTGACAATTATTGGAAGAGGTGCATTTGGA

99 K E T E Y M R L Q R H K M G V E D F E L L T I I G R G A F G

I

451 GAGGTGCGTCTTTGTAGAGAGAAGACCTCTAAAAGTGTATATGCAATGAAAAAGCTTAAGAAATCTGAAATGCTTCGTAGGGGCCAGGTG

129 E V R L C R E K T S K S V Y A M K K L K K S E M L R R G Q V

II

541 GAACACGTCAAAGCCGAAAGAAACCTCCTTGCAGAAGTCGATAGTGCGTACATAGTAAAGCTTTACTATTCTTTTCAAGATGATGAGTTC

159 E H V K A E R N L L A E V D S A Y I V K L Y Y S F Q D D E F

III IV

631 TTGTATCTCATCATGGAGTACCTTCCTGGTGGTGACATGATGACTTTGCTCATGCGCAAGGACACTCTGACAGAAGATGAAGCCAAATTT

189 L Y L I M E Y L P G G D M M T L L M R K D T L T E D E A K F

V

721 TACATCGCAGAAACTGTACTAGCAATAGAGTCCATTCACAAGCACAATTACATTCACAGGGATATCAAGCCAGATAATTTATTGTTAGAT

219 Y I A E T V L A I E S I H K H N Y I H R D I K P D N L L L D

VIa VIb

811 CTCAGTGGTCACTTGAAGCTTTCTGACTTTGGATTGTGCAAACCTTTGGATAGCAGTAATTTTCCAAATTTGAATGAACCGGATTATACA

249 L S G H L K L S D F G L C K P L D S S N F P N L N E P D Y T

VII

901 CCTGGAAAAGGTGCTAAACCTTTACCCGATAACACCAGTCGATTAACTAACTCTTCTGCACCGAAGCGTACGCAGCAGGAGCAGCTGTCA

279 P G K G A K P L P D N T S R L T N S S A P K R T Q Q E Q L S

991 CATTGGCAAAAGAACCGTCGGATGTTGGCGTATTCTACAGTTGGTACTCCTGATTACATTGCTCCAGAGGTTCTATTGAAGAAAGGATAT

309 H W Q K N R R M L A Y S T V G T P D Y I A P E V L L K K G Y

VIII

1081 GGAATGGAGTGTGACTGGTGGTCCCTTGGTGCTATCATGTATGAAATGCTAGTTGGTTATCCCCCATTTTATTCGGAGGATCCAATGTCG

339 G M E C D W W S L G A I M Y E M L V G Y P P F Y S E D P M S

IX

1171 ACCTGCAGAAAGATTGTGAACTGGAGAAGTCACCTGAAATTTCCTGAAGAGGCAAAGCTTTCTTCTGAAACTAAGGATCTCATTAGCAAA

369 T C R K I V N W R S H L K F P E E A K L S S E T K D L I S K

X

1261 CTTCTCTGTAATGTTGAGCAGAGACTTGGAACAAAAGGAGCCCATGAAATAAAAGCACATACATGGTTTAGAGGTGTCCAATGGGAAAAG

399 L L C N V E Q R L G T K G A H E I K A H T W F R G V Q W E K

IX

1351 TTGTATCAGATGAAAGCTGCTTTCATACCAGAAGTTAATGGCGAGTTGGATACTCAGAACTTTGAGAAATTTGAGGAGACTGGAGCACAA

429 L Y Q M K A A F I P E V N G E L D T Q N F E K F E E T G A Q

1441 GTTCAGAGTTCATCTAAGGCGGGTCCATGGAGAAAGATGCTTCCATCCAAGGATGCAAATTTTGTTGGATACACGTACAAGAACTTTGAA

459 V Q S S S K A G P W R K M L P S K D A N F V G Y T Y K N F E

1531 ATTGTGAATGACGATGAAGTTGCCGGGATTGCCGAGCTGAAGAAAAAGAGTTCCAAATCAAAACGGCCAACCATCAAGACATTGTTTGAG

489 I V N D D E V A G I A E L K K K S S K S K R P T I K T L F E

1621 AGCATGGATGAAGATGAACCTGTGCATGGCAGTTTCTTAAATATGTTGCCCCATAAGGAGGGACAACCTTCTTCTCACTCAAGCGCCCCA

519 S M D E D E P V H G S F L N M L P H K E G Q P S S H S S A P

1711 CCAGAACAATACCAACCTCGACGTAAATAGGATATGGAGGTTTTGAAAAGTAAACCACAGAAAAGTAAAAGAGAGGAAAAAAAACTGTTG

549 P E Q Y Q P R R K *

1801 CCTTTTGGTCTTGGTGTTACCCCGTCCAGCTTTTGTAAATCCTAACCGATGCTTTCTTTTTAGTGAATTTGTTAAAACAAAATCCATCCC

1891 CTGTCTCCAAAATAGTATGGTGTTTGATGTTAGCAGTGATGCCTGTTTGGTTCGCCGTATGTTGGTTTGACAGTCTCGGCGATGGGTCCC

1981 CGGCTATTACTGGCTACAGCATAAGTGTAAACTGTTACCAGCTCTGACGAGCATGTGTATTACAATTGAATTCACCTCTAAAAAAAAAAA

2071 AAAAAAA

Supplementary Fig. S4 Expression patterns of the wheat (*Triticum aestivum*) AGC kinase gene *TaAGC1* in wheat responding to exogenous hydrogen peroxide (H_2O_2) treatments. The reported expression levels of *TaAGC1* were relative to the control (0 h). Three biological replicates for each time point were averaged with the standard error of the means indicated. Asterisks indicate statistically significant variations calculated using the Student's *t*-test (*P < 0.05, **P < 0.01).

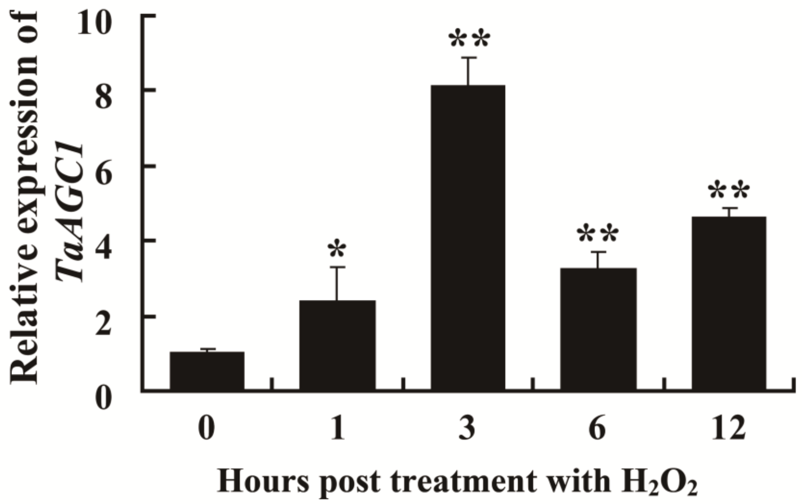

Supplementary Fig. S5 Analysis of hydrogen peroxide (H_2O_2) and superoxide anion (O_2^-) accumulation in wheat leaves. Leaves were harvested from wild-type wheat Yangmai 20 plants at the indicated day post inoculation (dpi) with *Rhizoctonia cerealis*, and then were stained with 3, 3-diaminobenzidine (DAB) and nitroblue tetrazolium (NBT) to detect H_2O_2 and O_2^-, respectively. Similar results were obtained from three independent replicates.

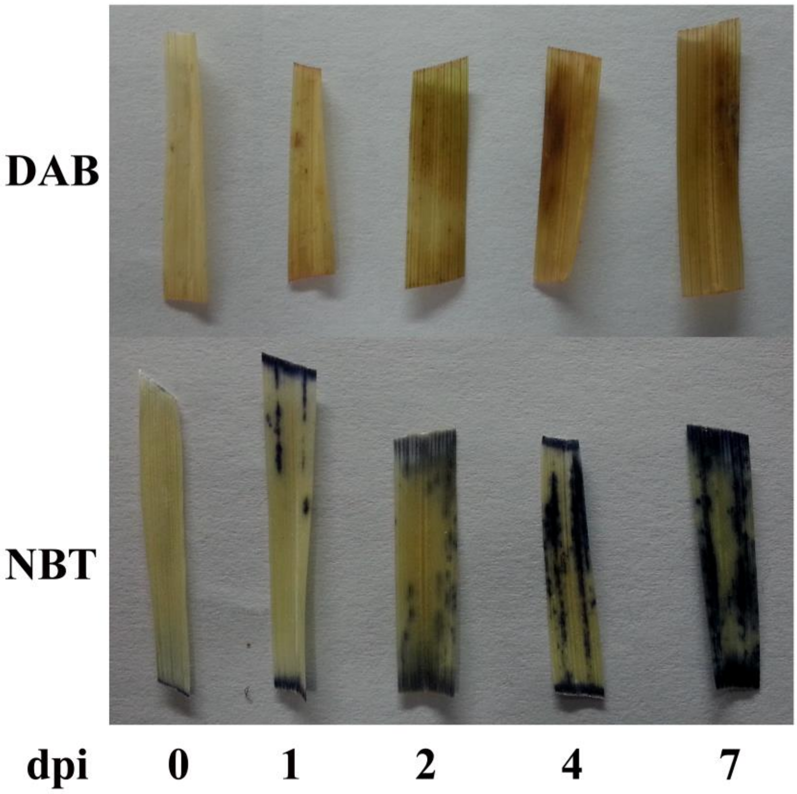

Supplementary Fig. S6 Transcriptional analysis of ROS- and defense-related genes in the wheat (*Triticum aestivum*) AGC kinase gene *TaAGC1*-overexpression and untransformated wheat Yangmai 20 (WT) plants under normal growth conditions. The reported transcript levels of the tested genes in the *TaAGC1*-overexpression lines were relative to those in WT plants. Statistically significant differences of *TaAGC1*-overexpressing wheat plants were compared with those of WT, and based on three biological replications (t-test; *P < 0.05, **P < 0.01). Bars indicate standard error of the mean (SE).

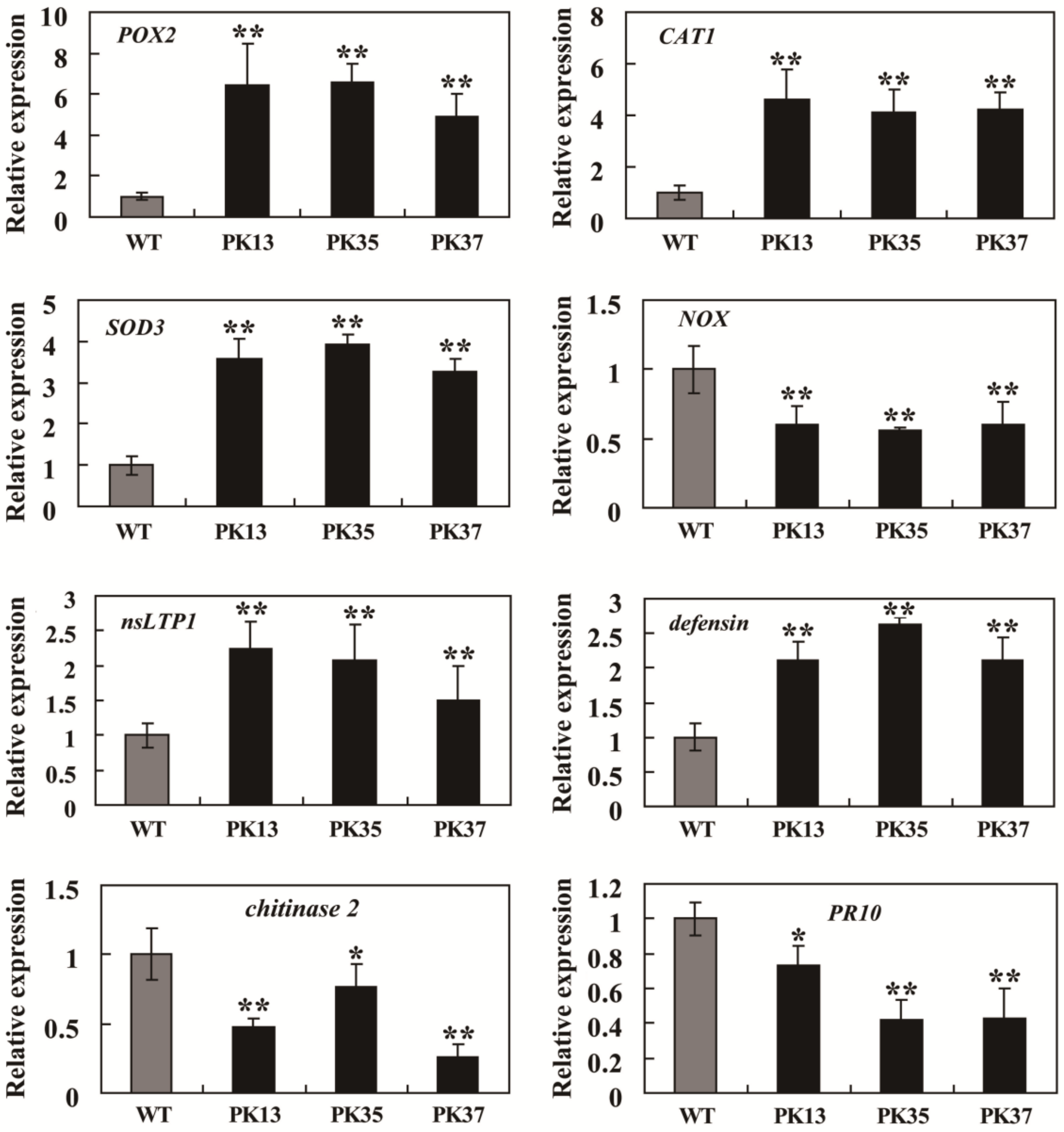

Supplementary Fig. S7 Transcription analysis of ROS-related and defense genes in the wheat (*Triticum aestivum*) AGC kinase gene *TaAGC1*- overexpressing and knock-down, as well as control wheat plants with *Rhizoctonia cerealis* inoculation for 40 d. The tested wheat samples include *TaAGC1*-overexpressing lines (PK13, PK35 and PK37), untransformed Yangmai 20 (WT) and *TaAGC1*-knock-down plants (BSMV:TaAGC1-1, -2 and -3) and BSMV-GFP- infected controls at 40 d post inoculation with *Rhizoctonia cerealis*. The reported transcript levels of the tested genes in the transgenic plants are relative to those in the WT plants. Statistically significant differences of *TaAGC1*-overexpression or *TaAGC1*-knock-down wheat plants were compared with WT or the controls, and based on three technical replications (*t*-test; *$P < 0.05$, **$P < 0.01$). Bars indicate standard error of the mean.

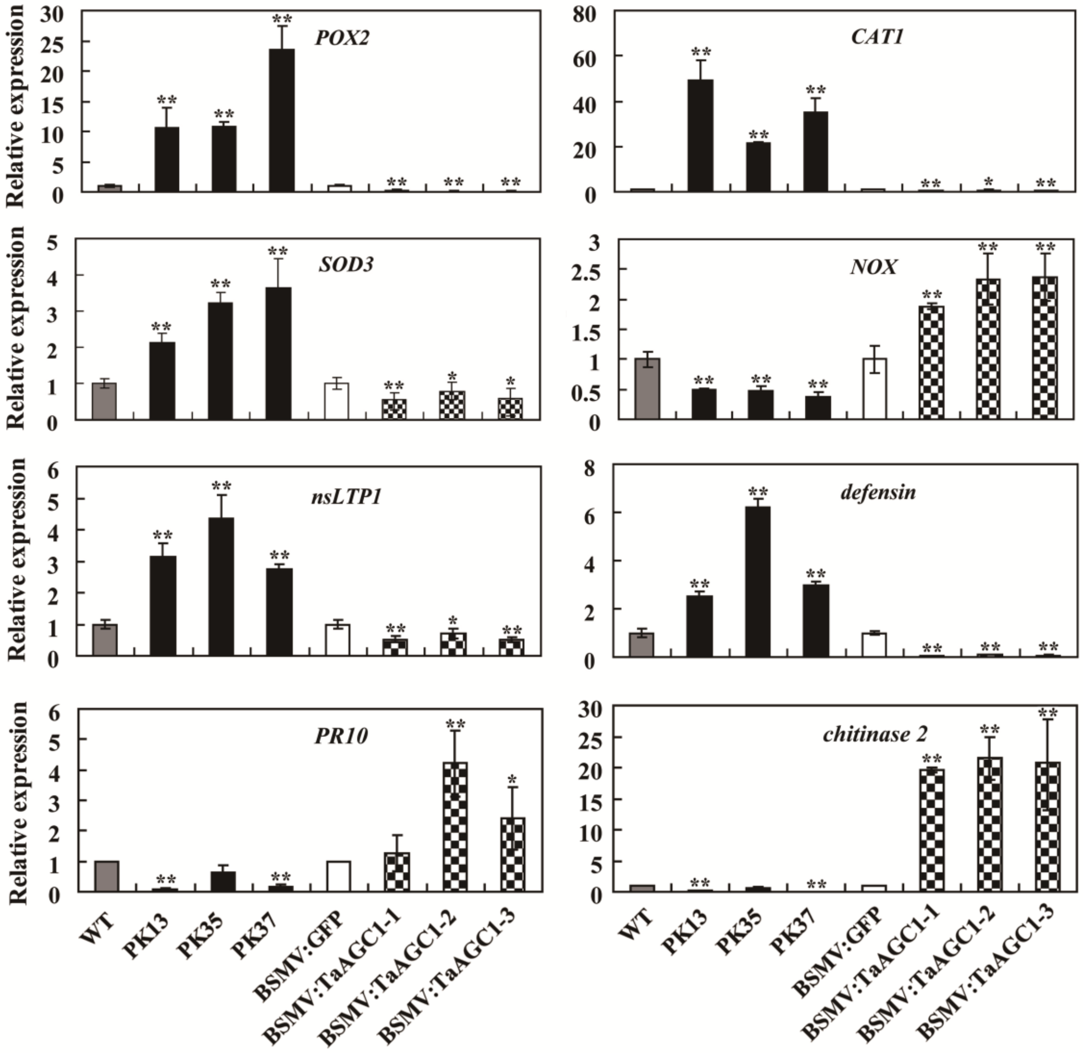

Supplementary Fig. S8 Transcription analysis of ROS-related and defense genes in the wild type wheat (*Triticum aestivum*) Yangmai 20 plants after *Rhizoctonia cerealis* inoculation for 7, 14, and 40 d. Total RNA was extracted from leaves of WT plants after *R. cerealis* inoculation for 0, 7, 14, and 40 d. The expression levels of those genes in the WT plants under normal conditions (0 d) were set to 1. Significant differences between *R. cerealis* inoculation (for 7, 14, and 40 d) and normal conditions (0 d) were derived from the results of three independent replications (*t*-test: *, P < 0.05; **, P < 0.01). Error bars indicate SE.

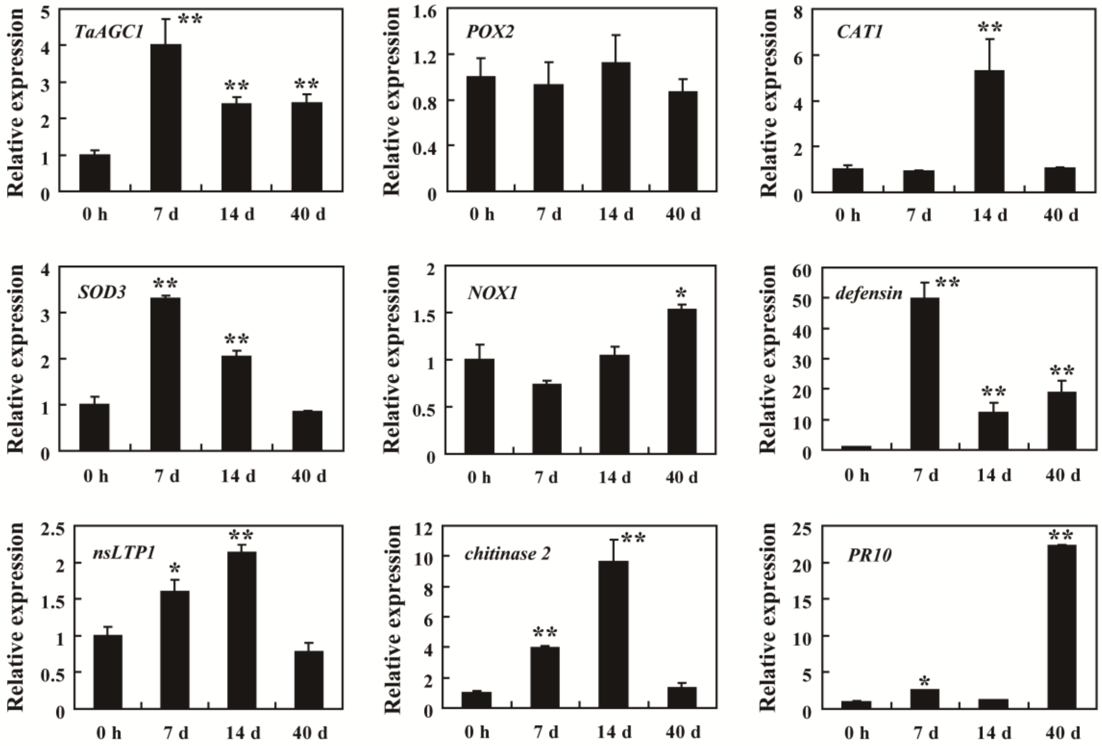

Supplementary Fig. S9 Alignment of 3’ terminal sequences of *TaAGC1* in resistant wheat line CI12633 and its homolog in susceptible wheat line Wenmai 6. The software DANMAN was used to perform the sequence alignment.

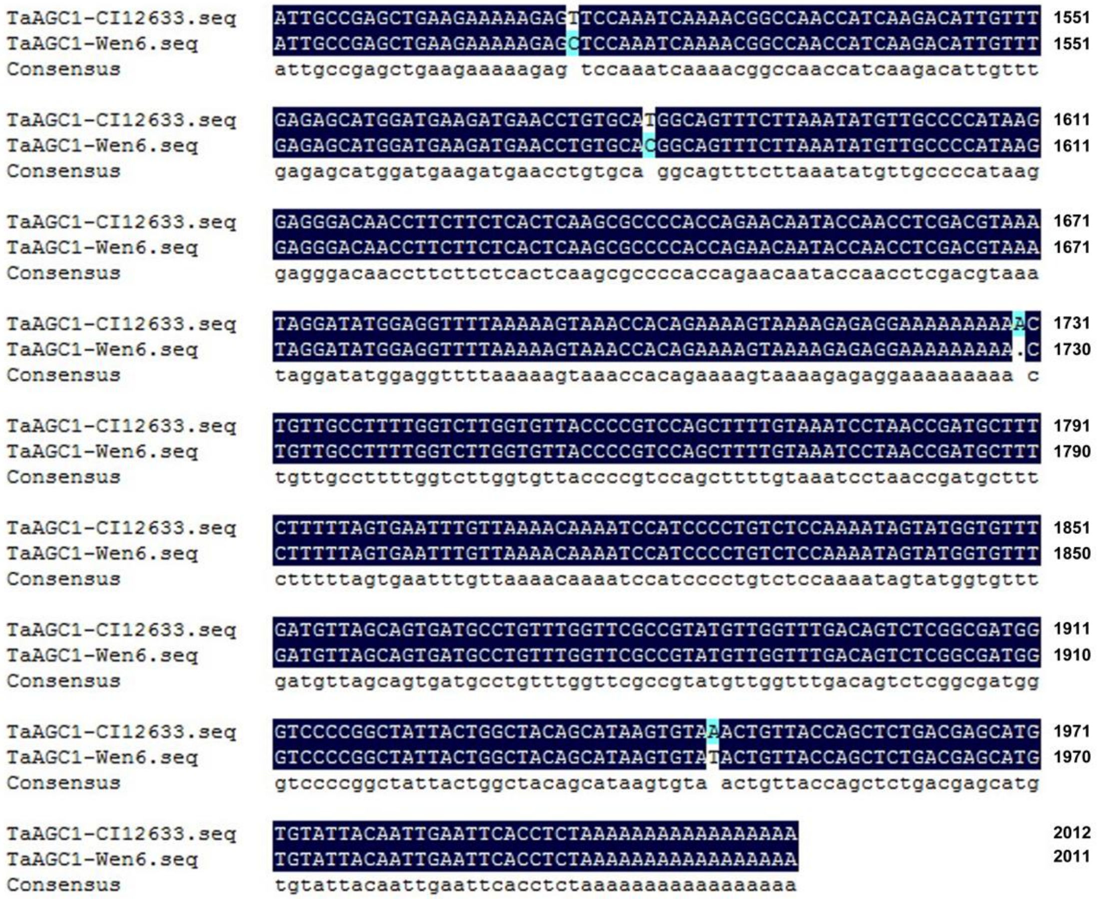

Supplementary Fig. S10 The powdery mildew symptoms of the wheat (*Triticum aestivum*) AGC kinase gene *TaAGC1*-overexpressing and knock-down, and control wheat plants.

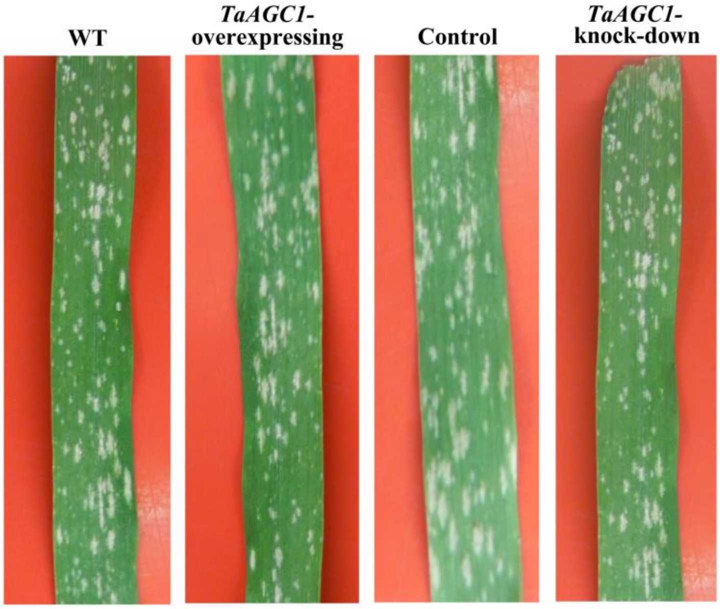

Supplementary Tables

Supplementary Table 1 *Rhizoctonia cerealis* responses of four wheat lines

Wheat lines	Sharp eyespot		Response degree
	Infection type	Disease index (%)	
CI12633	1.57	31.43	R
Shanhongmai	1.81	36.27	R
Yangmai 158	3.20	64.00	MS
Wenmai 6	3.67	73.33	S

Infection types (ITs) were categorized from 0 to 5 (i.e. “IT:0” = no lesion, “IT:1” = the lesion appeared on the sheaths rather than stems, “IT:2” = the width of the lesion < 50% of the infected stem perimeter, “IT:3” = the width of the lesion >50 and < 75% of the infected stem perimeter, “IT: 4”= the width of the disease lesion more than 75% of the infected stem perimeter, “IT: 5”= white spike or dead plant). The infection types were the average ITs of 30 plants for each line at 50 day post inoculation with *R. cerealis*. Disease index=$\{(0\times X_0+1\times X_1+2\times X_2+3\times X_3+4\times X_4+5\times X_5)/[(X_0+X_1+X_2+X_3+X_4+X_5)\times 5]\}\times 100$, where X_0-X_5 indicated plants with IT: 0-5. R, resistant; MS, moderately susceptible; S, susceptible.

Supplementary Table 2 Primers used in this study

Primer name	Accession number	Sequence of gene-specific primer	Usage
TaAGC1-3′-F1		5′-TAATGTTGAGCAGAGACTTGG-3′	3′ RACE for *TaCRK1*
TaAGC1-3′-F2		5′-ATGAAGTTGCCGGGATTGC--3′	
TaAGC1-ORF-F1		5′-GCATCGGGTTCTACGGAG-3′	Amplification of full-length cDNA of *TaCRK1*
TaAGC1-ORF-R1		5′-GAGACTGTCAAACCAACATACG-3′	
TaAGC1-ORF-F2		5′-CGGCTGCTGGATTGGT-3′	
TaAGC1-ORF-R2		5′-CGGCGAACCAAACAGG-3′	
TaAGC1-1584F		5′-ATGAAGTTGCCGGGATTGC-3′	Detection of transformed *TaAGC1*
tNOS-L23		5′-ATGTATAATTGCGGGACTCTAAT-3′	
TaAGC1-Q-F		5′-ATGAAGTTGCCGGGATTGC-3′	qRT-PCR for *TaCRK1* transcript
TaAGC1-Q-R	KJ686386	5′-CGGCGAACCAAACAGG-3′	
TaAGC1-GST-F		5′-CGCGAATTCATGGATTCCGCGAGAAGT-3′	Construction of vector pGST-TaAGC1
TaAGC1-GST-R		5′-CGTCTCGAGCTATTTACGTCGAGGTTG-3′	
TaAGC1-γ-F		5′-TACGCTAGCATGAACCTGTGCATGGCAGT-3′	Construction of vector γ-TaAGC1
TaAGC1-γ-R		5′-TACGCTAGCCAGGCATCACTGCTAACATCA-3′	
TaAGC1-p25-F		5′-GAACTAGTATGGATTCCGCGAGAAGT-3′	Construction of vector pMyc-TaAGC1
TaAGC1-p25-R		5′-GCGAGCTCCTATTTACGTCGAGGTTG-3′	
D239A-F		5′-GCTATCAAGCCAGATAATTTAT-3′	Construction of vector pGST-D239A
D239A-R		5′-CCTGTGAATGTAATTGTGCTTG-3′	
BSMV-CP-F	JF803284	5′-TGACTGCTAAGGGTGGAGGA-3′	Detection of BSMV virus
BSMV-CP-R		5′-CGGTTGAACATCACGAAGAGT-3′	
TaActin-F	BE425627	5′-CACTGGAATGGTCAAGGCTG-3′	Internal control for qRT-PCR
TaActin-R		5′-CTCCATGTCATCCCAGTTG-3′	
POX2-F	X85228	5′- AGGGGCTTCGGCGTCATC-3′	qRT-PCR for *POX2* transcript
POX2-R		5′- TTGGGCGTCGTCGTGTCC-3′	
TaCAT1-F	GU984379	5′-CAAGGGCTTCTTCGAGGTCAC-3′	qRT-PCR for *TaCAT1* transcript
TaCAT1-R		5′- TGTAGAAGGTCCACTCCGGGTAG-3′	
TaNOX-F	AY561153	5′-ATGTTCGGCAACTTGGTGACT-3′	qRT-PCR for *TaNOX* transcript
TaNOX-R		5′- CGTCTGCTCTAAGAAGACCACTTTT-3′	
nsLTP1-F	TC411506	5′-ATGCGGGTTGGCGTGAAG-3′	qRT-PCR for

nsLTP1-R		5′-TGTTGCGGTGGTAGGTTGTTG-3′	*nsLTP1* transcript
Defensin-F	CA630387	5′- ATGTCCGTGCCTTTTGCTA-3′	qRT-PCR for *defensin* transcript
Defensin-R		5′- CCAAACTACCGAGTCCCCG-3′	
SOD3-F	U72212	5′- CAGAGGGTGCTGCTTTACAA-3′	qRT-PCR for *SOD3* transcript
SOD3-R		5′- CCAACAGCGGGAAACTCAA-3′	
Chit2-F	TC426538	5’-TTCTGGATGACGGCACAAG-3’	qRT-PCR for *Chit2* transcript
Chit2-R		5’-CCTTAGTGTGACCAGTCGTTTT-3’	
PR10-F	CA613496	5’- CGTGGAGGTAAACGATGAG-3’	qRT-PCR for *PR10* transcript
PR10-R		5’- GCTAAGTGTCCGGGGTAAT-3’	
